# Advanced polymer hydrogels that promote diabetic ulcer healing: mechanisms, classifications, and medical applications

**DOI:** 10.1186/s40824-023-00379-6

**Published:** 2023-04-26

**Authors:** Yamei Xu, Qiyuan Hu, Zongyun Wei, Yi Ou, Youde Cao, Hang Zhou, Mengna Wang, Kexiao Yu, Bing Liang

**Affiliations:** 1 Department of Pathology, College of Basic Medicine, Chongqing Medical University, 1 Yixueyuan Road, Yuzhong Distinct, Chongqing, 400016 P.R. China; 2 Molecular Medicine Diagnostic and Testing Center, Chongqing Medical University, 1 Yixueyuan Road, Yuzhong Distinct, Chongqing, 400016 P.R. China; 3 Department of Pathology, the First Affiliated Hospital of Chongqing Medical University, 1 Youyi Road, Yuzhong Distinct, Chongqing, 400042 P.R. China; 4 Department of Orthopedics, Chongqing Traditional Chinese Medicine Hospital, No. 6 Panxi Seventh Branch Road, Jiangbei District, Chongqing, 400021 P.R. China; 5 Institute of Ultrasound Imaging of Chongqing Medical University, 1 Yixueyuan Road, Yuzhong Distinct, Chongqing, 400016 P.R. China

**Keywords:** Diabetic ulcers, Hydrogels, Wound healing, Polymers, Mechanism

## Abstract

Diabetic ulcers (DUs) are one of the most serious complications of diabetes mellitus. The application of a functional dressing is a crucial step in DU treatment and is associated with the patient's recovery and prognosis. However, traditional dressings with a simple structure and a single function cannot meet clinical requirements. Therefore, researchers have turned their attention to advanced polymer dressings and hydrogels to solve the therapeutic bottleneck of DU treatment. Hydrogels are a class of gels with a three-dimensional network structure that have good moisturizing properties and permeability and promote autolytic debridement and material exchange. Moreover, hydrogels mimic the natural environment of the extracellular matrix, providing suitable surroundings for cell proliferation. Thus, hydrogels with different mechanical strengths and biological properties have been extensively explored as DU dressing platforms. In this review, we define different types of hydrogels and elaborate the mechanisms by which they repair DUs. Moreover, we summarize the pathological process of DUs and review various additives used for their treatment. Finally, we examine the limitations and obstacles that exist in the development of the clinically relevant applications of these appealing technologies.

**Graphical Abstract:**

This review defines different types of hydrogels and carefully elaborate the mechanisms by which they repair diabetic ulcers (DUs), summarizes the pathological process of DUs, and reviews various bioactivators used for their treatment.

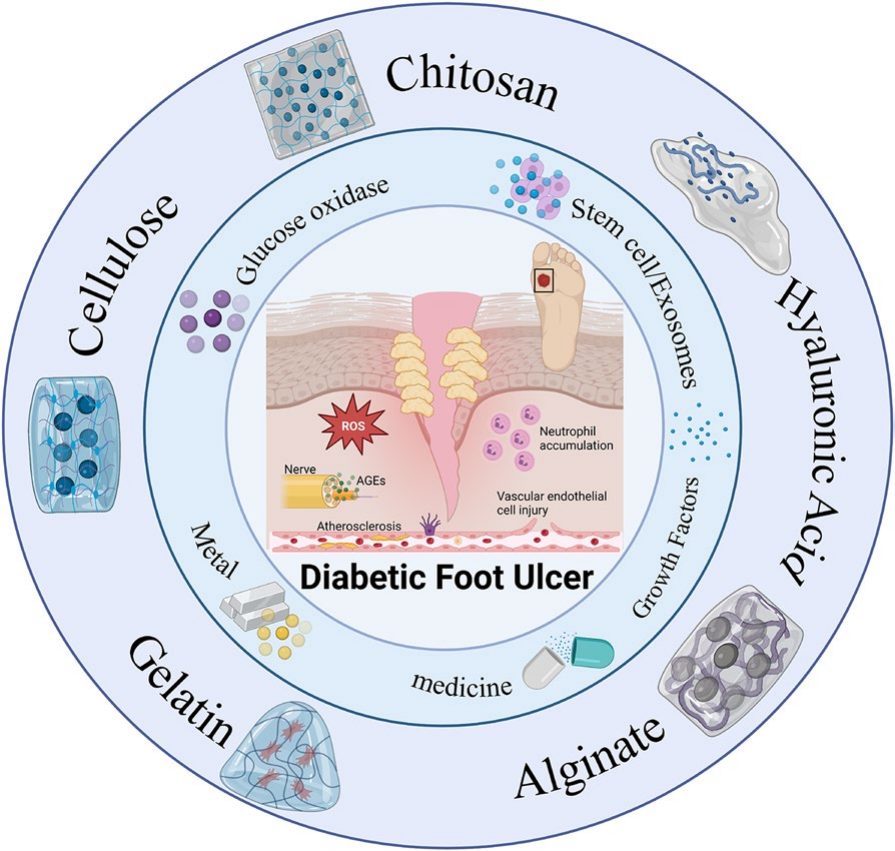

## Introduction

Diabetes mellitus is a chronic, long-term worldwide epidemic disease [1, 2]. According to data published by the American Diabetes Association (ADA) and epidemiological surveys, the global economic burden of diabetes will reach $2.1 trillion by 2030, accounting for 2.2% of the worldwide GDP [3]. Severe disabilities are associated with diabetes, including blindness, diabetic retinopathy, end-stage renal failure, and lower extremity amputations [4]. The psychological and physical effects on patients are immense and they are difficult to heal.

Diabetic ulcers (DUs) are one of the most serious complications of diabetes mellitus, in particular diabetic foot ulcers (DFUs) [5], and their pathophysiological mechanisms are exceptionally complex and include the following: 1. hyperglycemia-mediated impairment of endothelial cell function and development of vascular lesions and peripheral neuropathy [6–8]; 2. a prolonged chronic inflammatory response brought about by the release of cytokines and transformation failure of M1 macrophages [9–11]; 3. increased oxidative stress due to sustained reactive oxygen species (ROS) production by neutrophils and macrophages [12]; 4. skin barrier disruption caused by the colonization of multiple microbes, etc. [13]. A pilot study showed that Staphylococcus and Streptococcus were the most common colonizing strains in mild to moderate DFU trauma [14]. These bacteria can secrete proteolytic factors that damage the skin barrier, which subsequently leads to long-lasting wounds and even amputation. According to previous reports, 140 thousand amputations that take place in the US are related to diabetes [15]. For DFUs, due to increased risk of limb amputations of the lower extremities, DFUs are associated with approximately 50% mortality within 5 years of amputation [16]. However, after amputation, the wound becomes larger and more difficult to heal, which then leads to increased incidence of amputation in a vicious cycle that undoubtedly increases the psychological and economic burden of DU patients. Therefore, based on the societal development, social needs and changes in the disease spectrum, both patients and doctors have new requirements for the treatment of DUs that include not only early trauma intervention but also preservation of the affected limbs to the greatest extent possible and reductions in the psychological pressure and economic burden of patients.

The standard treatment for DUs includes debridement, antibiotics to control the infection, foot decompression, and closure with a dressing [17]. Clinicians have relied on this regimen to relieve pain and extend the lives of many DU patients over the past few decades, but the number of true cures for DUs remains unsatisfactory, and most treatment regimens end with amputation or death. Therefore, doctors and scientists are looking for new treatments to extend patients' lives, preserve their affected limbs and improve their quality of life. Dressing closure is a crucial part of the DU treatment process. A good dressing not only covers the wound and absorbs exudate but also prevents microbial invasion, mimics the natural extracellular matrix (ECM) environment, accelerates wound healing, tries to heal the ulcer at an early stage, and reduces the risk of amputation or death of the patient at a later stage.

At present, there are two main concepts in academia in the field of wound healing, dry healing and wet healing [18], and medical skimmed cotton gauze and petroleum jelly gauze prepared according to the traditional dry healing concept are the most widely used skin wound dressings in clinical practice. However, dry dressings tend to dehydrate and crust the wound, which is not conducive to epithelial cell spreading, and cannot isolate bacterial invasion [19, 20]. To solve the above problems of traditional dry dressings, a series of new wet healing dressings have been developed, including hydrogels, films, hydrocolloids, foams, etc. [9, 21]. Among them, hydrogels are of great interest due to their following advantages. First, hydrogels have good biocompatibility. This allows them to mimic the natural ECM environment and provide a suitable environment for cell proliferation [22]. Second, hydrogels possess excellent water retention properties and permeability. Due to the presence of a crosslinked network, the water content of hydrogels can reach up to 96% [23], which can allow hydration of the environment and keep the wound moist. Moreover, the existence of a porous network structure provides a good environment for gas exchange and nutrient transport. Third, due to their unique structural properties, hydrogels do not cause secondary mechanical damage [24]. The granulation tissue in wounds has a propensity to grow into traditional dry dressings in the form of a mesh, tending to adhere to the dressing when it is being changed and cause secondary mechanical damage. Additionally, hydrogels promote the debridement and absorption of exudate well, especially in terms of necrotic tissue autolysis [18]. When in contact with tissues, hydrogels help absorb water from the tissues and transfer into the dressing through repeated hydration, effectively drying the wound. Moreover, hydrogels can be used as vehicles for carrying therapeutic drugs [4, 25]. In recent years, the biological functions and mechanical properties of hydrogels have been enhanced by the introduction of biologically active factors to achieve precise and extended regulation of the microenvironment of chronic ulcer wounds [26].

In this review, we review the pathogenic process of DUs, define the different types of natural polymeric hydrogels used for DU therapy, and elaborate on the mechanisms by which hydrogels repair these wounds. In addition, we summarize the bioactive substances used for DU treatment and discuss their advantages and disadvantages and propose future directions. Finally, we debate the challenges and prospects for the further clinical translation of hydrogels. We hope to provide novel ideas and a theoretical basis for regenerative medicine and tissue engineering research.

## The pathological process of DUs

The DU healing process does not follow the general wound healing pattern (hemostasis, inflammation, proliferation, remodeling) and is often prolonged due to the chronic and persistent inflammatory phase, which is the result of the interaction of multiple risk factors. Hyperglycemia, the initiating factor of DUs, leads to vasculopathy and neuropathy; and under the combined effects of the above lesions, diabetic wounds initially form. Moreover, the combination of persistent inflammation, excessive oxidative stress, and infection leads to the gradual deterioration of diabetic wounds to eventually form chronic nonhealing wounds, i.e., DUs.

### Hyperglycemia: the factor that initiates the DU microenvironment

Hyperglycemia is a factor that initiates abnormal metabolic pathways in patients with DUs and is the main cause of prolonged wound healing, which presents as two main types of damage: angiopathy and neuropathy. The mechanisms underlying the chronic complications of hyperglycemia have not been fully elucidated. Currently, many researchers have focused on four pathways: the polyol pathway, protein kinase C (PKC) pathway, hexosamine biosynthesis pathway (HBP), and the accumulation of advanced glycosylation end products (AGEs)

#### Polyol pathway

**Fig. 1:**
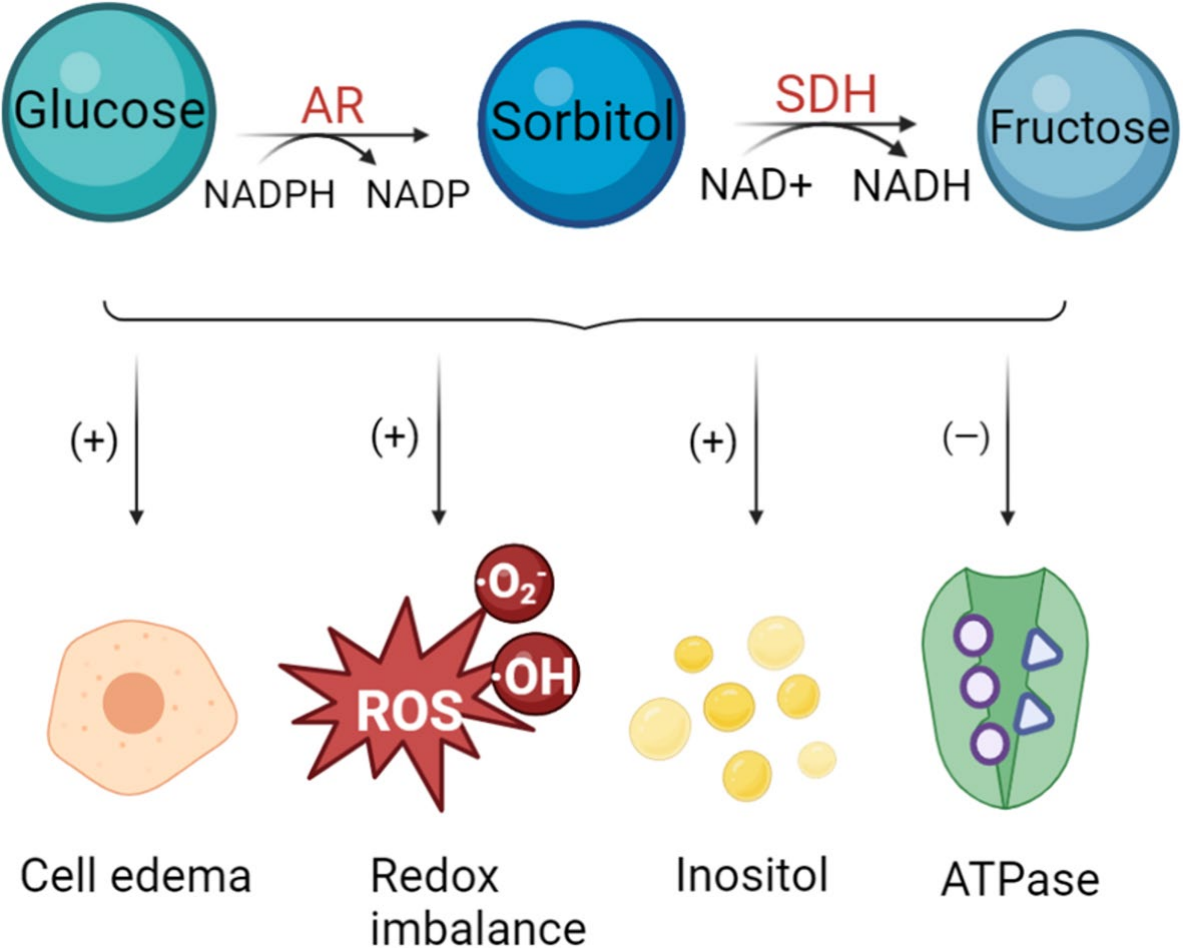
Two major reactions in the polyol pathway and their effects on cells in high glucose environments

The polyol pathway involves two enzymatic reactions [27–29] (Fig. 1). First, aldose reductase (AR) consumes NADPH to catalyze the production of sorbitol from glucose, then sorbitol dehydrogenase (SDH) converts sorbitol into fructose using NAD^+^ as a coenzyme. Under normal conditions, only a small amount of glucose enters the polyol pathway [30]. However, in diabetes, the increase in blood glucose concentration raises the activity of the rate-limiting enzyme AR [31], causing an influx of glucose into the polyol pathway.

Various hypotheses have been proposed to explain the tissue and cell injuries caused by increased flux of glucose into the polyol pathway. Osmotic damage was one of the first hypotheses proposed [32, 33]. Due to the increase in AR activity, large amounts of sorbitol accumulate in cells, leading to intracellular hyperosmolarity, which in turn causes cellular edema and degeneration. Elena Berrone et al. demonstrated the increases in AR expression, AR activity and sorbitol levels in human endothelial cells cultured under high glucose conditions [34]. However, this explanation does not seem to be widely applicable to other tissues [35].

Therefore, the theory of oxidative stress was proposed [12]. The 2 reactions above consume NADPH and NAD^+^, causing a redox imbalance and interfering with normal metabolism [36, 37]. Notably, an abnormal oxidative stress level also activates the PKC pathway and increases the accumulation of AGEs [38]. In addition, the accumulation of sorbitol and the loss of coenzymes cause a deficiency in intracellular inositol and a decrease in Na^+^-K^+^-ATPase activity, resulting in cell damage [39, 40].

#### PKC pathway

**Fig. 2:**
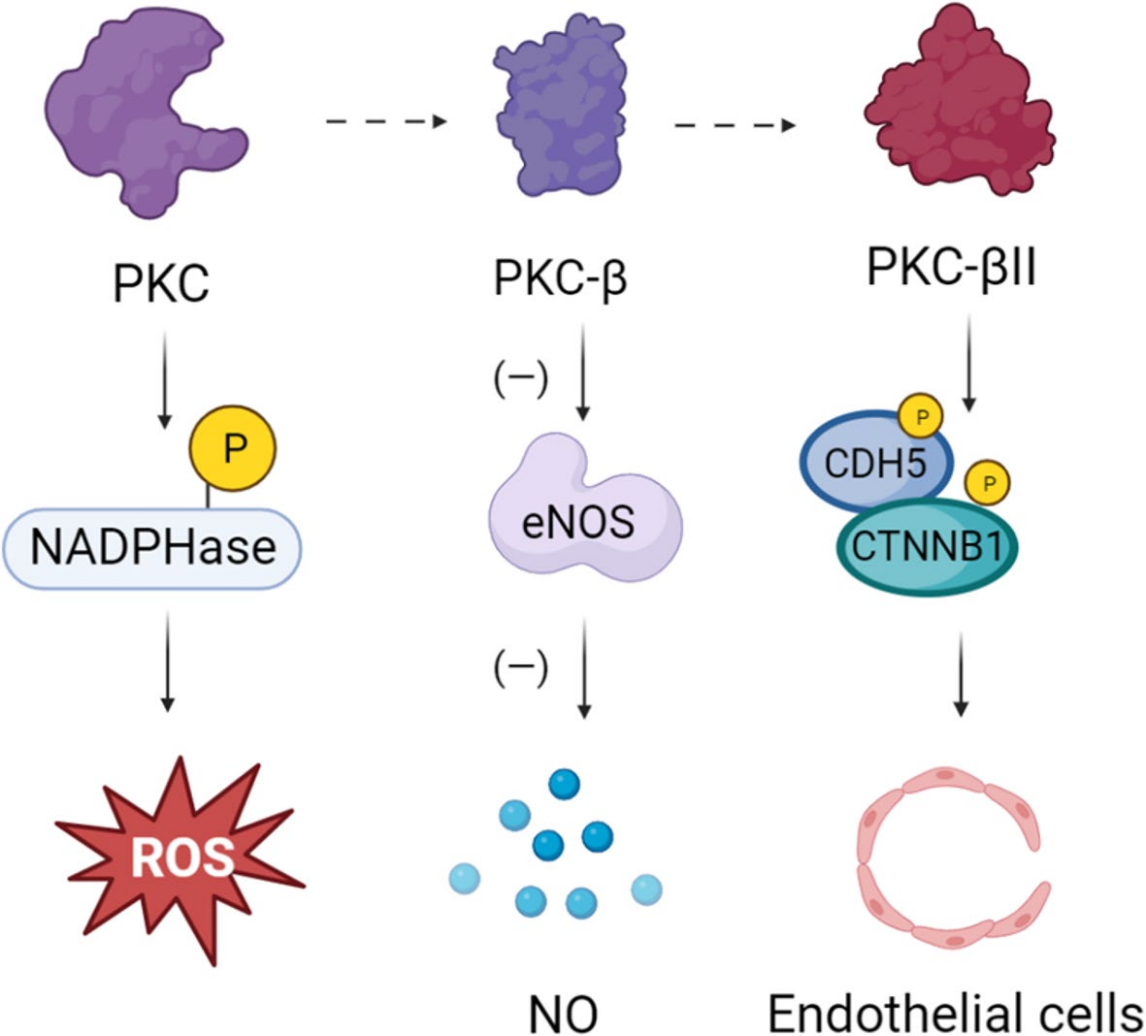
Abnormal activation of PKC pathway leads to REDOX imbalance, NO reduction, and endothelial cell injury

PKC is a member of the serine/threonine kinase family and can be classified into three subgroups, including classical/conventional PKCs (cPKCs), novel PKCs (nPKCs), and atypical PKCs (aPKCs), depending on the mode of activation [41]. As a commonly expressed enzyme, different PKC isoforms are activated during the progression of diseases in different tissues and organs [42]. Among them, activation of PKC-β plays an important role in vascular diseases [38, 43]. Diabetic endotheliopathy is considered to be one of the common bases of microvascular and macrovascular lesions, which mainly manifest as endothelium-dependent vasodilatory dysfunction [44]. Nitrogen monoxide is the primary endogenous vasodilator and is synthesized in endothelial cells through catalysis by endothelial-type nitric oxide synthase (eNOS) [45]. Tabit et al. reported that PKC-β expression is higher in endothelial cells of type 2 diabetes patients and is involved in the insulin-resistant impairment of eNOS activation, which leads to reduced NO production and vasoconstriction and subsequently exacerbates local ischemia in DUs [46]. In addition, PKC-βII is associated with endothelial cell permeability, and its activation leads to phosphorylation of the tight junction proteins VE-calmodulin (CDH5) and β-linked protein (CTNNB1) [47, 48], ultimately disrupting interendothelial cell junctions. Notably, activation of the PKC pathway is also related to oxidative stress imbalance, which leads to the direct phosphorylation of the NADPH oxidase subunit to promote ROS production in vascular cells [49], thus affecting endothelial cell function (Fig. 2).

#### Hexosamine biosynthesis pathway

**Fig. 3:**
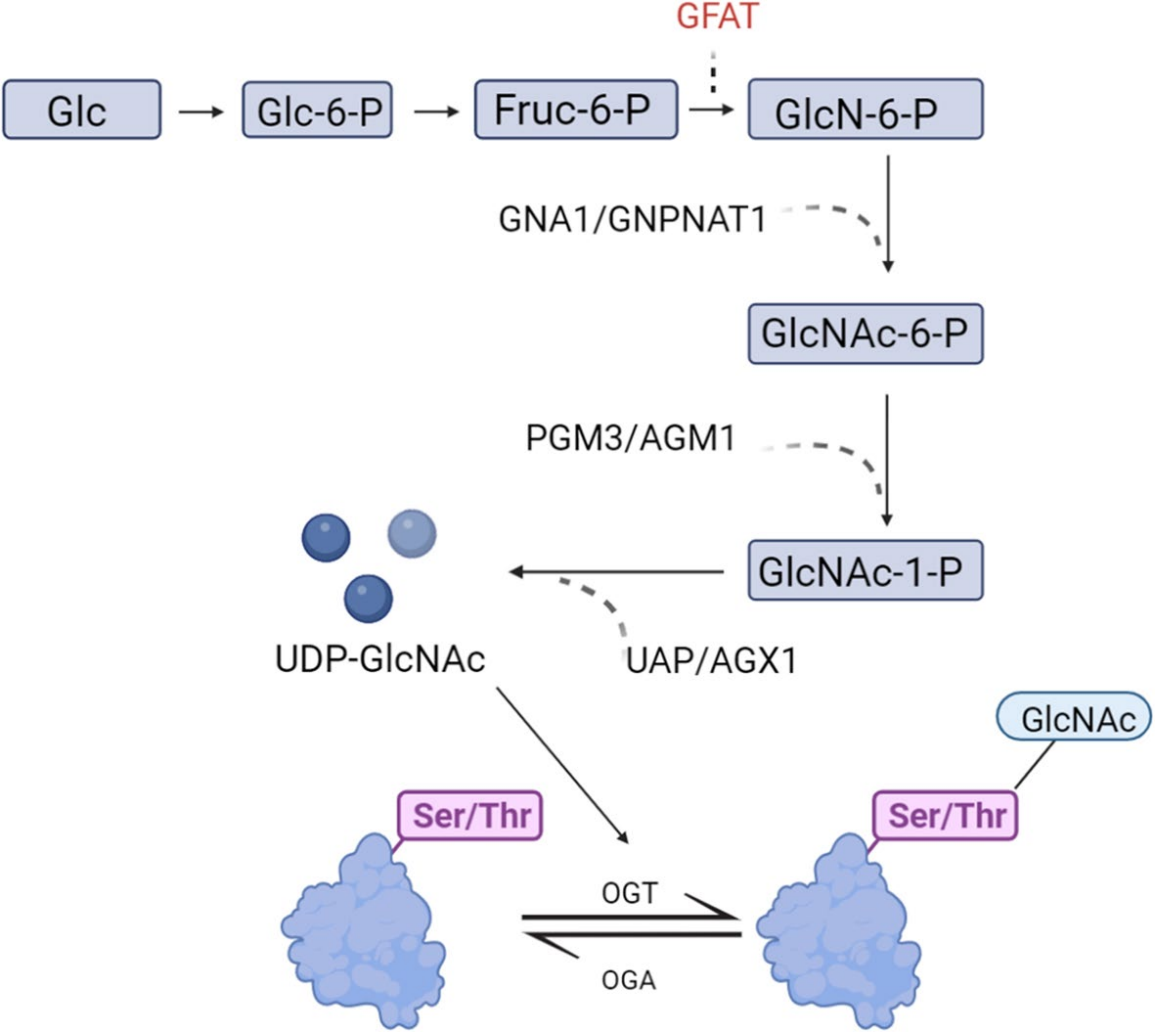
The hexosamine pathway

N-Acetylglucosamine (O-GlcNAc) glycosylation (also known as O-GlcNAcylation) is a highly reversible, dynamic posttranslational modification that regulates protein function by GlcNAc moieties to serine/threonine residues in a polypeptide chain [50]**.** In humans, this process is regulated by a pair of highly conserved enzymes: O-GlcNAc transferase (OGT) and O-GlcNAcase (OGA). OGT catalyzes the reaction between UDP-GlcNAc and the hydroxyl groups of the above two amino acid residues to form amide bonds to participate in the modification process, while OGA is responsible for reversing this protein modification [51] (Fig. 3).

Aberrant O-GlcNAcylation affects many downstream proteins [52]. For example, when the osteogenic transcription factor Runx2 is expressed in vascular cells, it has been shown to promote calcification and matrix protein production [53]. Heath et al. treated mouse vascular smooth muscle cells (VSMCs) with Thiamet G, a potent inhibitor of OGA, and upregulated Runx2 expression, as evidenced by the increased aortic O-GlcNAcylation and vascular calcification in a diabetic mouse model [54]. The HBP has been shown to be significantly associated with persistent chronic inflammation [55]. The transcription factor NF-κB plays a key role in the inflammatory and immune responses of cells. It has been reported that O-GlcNAcylation at threonine-352 of the NF-κB p65 subunit in rat VSMCs inhibits the interaction between NF-κB and IκB so that NF-κB can enter the nucleus, which explains why NF-κB is continuously activated in diabetes and mediates the generation of chronic inflammation to a certain extent [56].

#### AGE accumulation

**Fig. 4:**
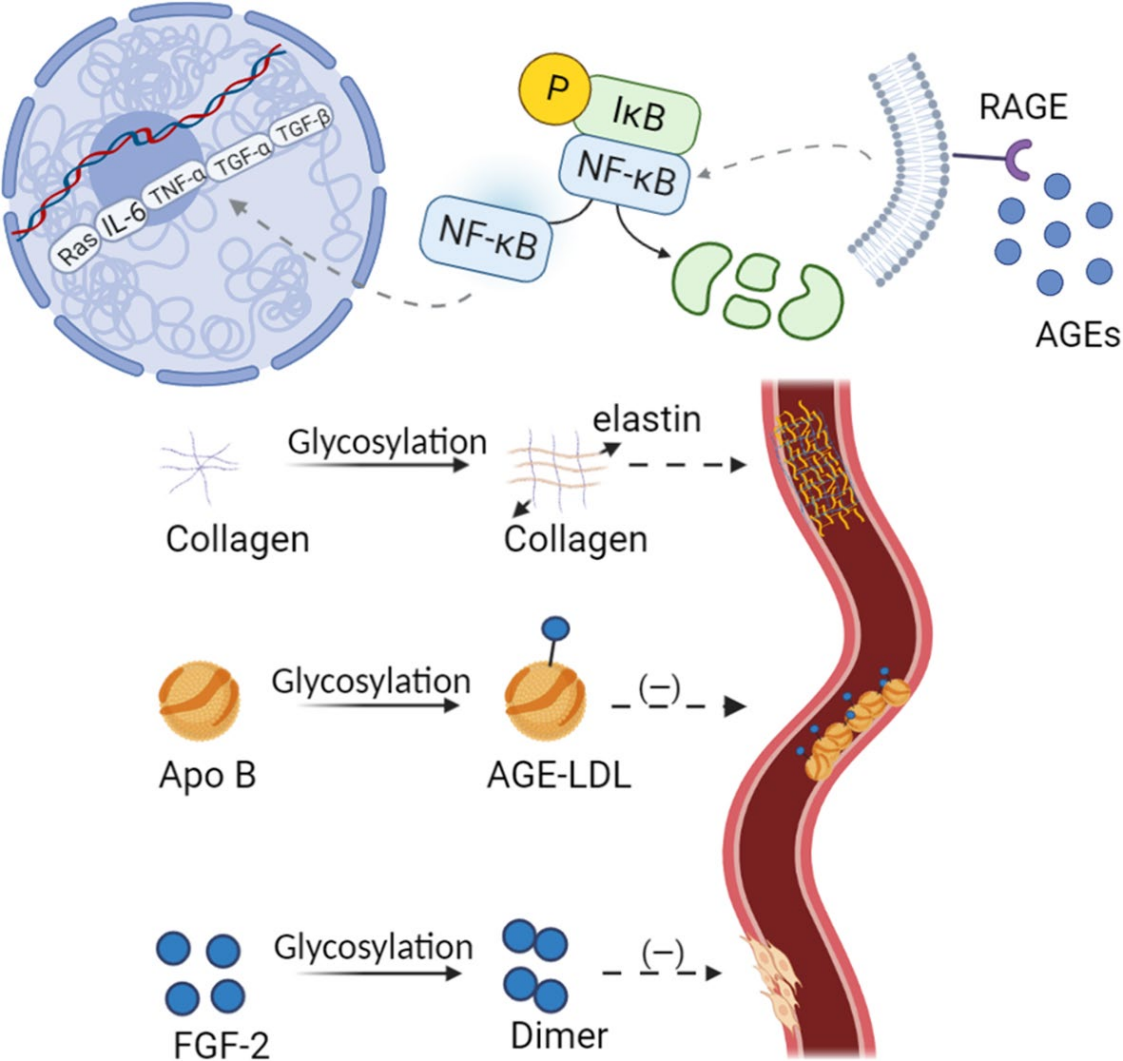
Non-enzymatic glycosylation of collagen, ApoB and FGF-2 impeded wound healing and AGEs bind to receptors leading to abnormal activation of the NF-κB pathway and persistent inflammation

AGEs are stable, covalent adducts generated by the nonenzymatic glycosylation of macromolecules such as proteins, nucleic acids, and lipids with glucose or other reducing sugars that are present in the body in excess [31, 43]. AGE-induced tissue and cell damage is mainly achieved through two pathophysiological mechanisms (Fig. 4).

First, proteins modified by nonenzymatic glycosylation reactions undergo structural and functional alterations. Because AGEs take considerable time to form, nonenzymatic glycosylation reactions often occur in long-lived proteins (e.g., collagen) in vivo. Glycosylation creates crosslinks between collagen and elastin, and these crosslinked products not only resist protease hydrolysis but also inhibit matrix metalloproteinase (MMP) expression and activation, leading to collagen accumulation and fibrosis [57]. In addition, nonenzymatic glycosylation causes abnormal lipid metabolism and atherosclerosis. Apolipoprotein B (Apo B) in low-density lipoprotein (LDL) is modified by nonenzymatic glycosylation to form AGE-LDL, which prevents LDL from binding to its receptor to mediate lipid clearance [58]. In parallel, fibroblast growth factor-2 (FGF-2) can be nonenzymatically glycosylated in a high-glucose environment, leading to dimer formation, which reduces the ability of FGF-2 to bind to receptors and affects fibroblast proliferation and collagen synthesis [59].

Second, the binding of AGEs to supracellular receptors causes abnormal signaling pathway activation. Receptor for advanced glycosylation end products (RAGE) is the most important receptor for AGEs. The AGE-RAGE interaction activates several signaling pathways, such as Jak/Stat, mitogen-activated protein kinases (MAPKs) (e.g., p38, extracellular regulated (ERK)-1/2) and c-Jun N-terminal kinase (JNK) [60–65]. Among them, NF-κB is the main downstream target of several of the signaling pathways mentioned above. Upon activation, NF-κB increases the activity of certain enzymes (e.g., NADPH oxidase) while upregulating the expression of proinflammatory cytokines (e.g., IL-6, TNF-α, TGF-α, TGF-β) and vascular adhesion molecules, thus inducing inflammation and oxidative stress and facilitating the development of DFUs. Jin et al. incubated macrophages with AGEs and showed that AGEs significantly promoted macrophage expression of IL-6 and TNF-β by activating the NF-κB pathway [66]. Unfortunately, accumulated AGEs are difficult to clear in vivo, and even if hyperglycemia is reversed, the toxic effects of AGEs can continue to exist [67, 68].

### Main manifestations of DUs: angiopathy and neuropathy

Diabetic vasculopathy is a common complication of diabetes mellitus, and in a prospective cross-sectional study by Ahmed Azhar, the incidence of vasculopathy in patients with DFU syndrome was as high as 43.87% [69]. The pathophysiological theme of peripheral arterial disease (PAD) in the progression of DU is atherosclerosis, which occurs mainly in the arteries of the lower limbs, especially the dorsalis pedis artery [70, 71]. This may be due to the long, thick arteries of the lower extremities, which are susceptible to experience high blood pressure and have a greater chance of intimal damage. PAD is the result of multiple interrelated pathogenic factors induced by hyperglycemia and initiated through the abovementioned 4 pathways and includes endothelial and VSMC dysfunction, inflammation, and platelet aggregation. In addition, diabetic atherosclerosis is often associated with hyperinsulinemia and disorders of lipid metabolism. Supraphysiological doses of insulin can increase cholesterol synthesis by promoting the dephosphorylation of HMG CoA in addition to inhibiting fat mobilization by inhibiting adenylate cyclase. Reduced fat metabolism in the body also causes the deposition of LDL and very low-density lipoprotein (VLDL) in the subendothelial matrices of arteries, which are phagocytosed by smooth muscle and macrophages to form foam cells to induce and exacerbate atherosclerosis [72]. Type 2 diabetes patients often have endogenous hyperinsulinemia due to insulin resistance, while type 1 diabetes patients often experience hyperinsulinemia due to exogenous insulin therapy even though they do not secrete insulin endogenously. Therefore, both type 1 and type 2 diabetes patients can develop atherosclerosis in large blood vessels through hyperinsulinemia.

Diabetic peripheral neuropathy is also a common complication of diabetes mellitus and often presents clinically as symmetric pain with a glove or garter-like distribution in the limbs [73]. The pathogenesis of diabetic peripheral neuropathy is closely related to the four pathways and vascular lesions described above. AR was shown to be distributed in Schwann cells as evidenced by early peripheral nerve fiber demyelination and axonal degeneration, Schwann cell proliferation, and immunohistochemistry, suggesting that peripheral nerve tissue can cause large amounts of sorbitol and fructose to accumulate intracellularly through the polyol pathway, leading to nerve cell swelling, degeneration, and necrosis [74]. In addition, biopsies of the gastrocnemius and femoral nerves in type 2 diabetes patients revealed significant increases in the deposition of AGEs in the axons and myelin sheaths, and these AGEs could bind to RAGE highly expressed on the micro-vessels at the nerve bundle membrane, nerve inner membrane, and nerve outer membrane. This leads to activation of the NF-κB pathway and an increase in the release of inflammatory factors such as IL-6 and IL-8, which triggers an inflammatory response that interferes with the normal functions of blood vessels and leads to inflammatory vascular neuropathy [75]. Of note, the diabetic vasculature is stimulated by the long-term presence of high glucose concentrations. This leads to atherosclerosis and luminal narrowing, which results in an insufficient blood supply to the distal peripheral circulation, hypoperfusion and intimal ischemia in the nerve trophoblastic vessels, further aggravating nerve degeneration and necrosis [76].

### DUs persist in a state of chronic inflammation and oxidative stress

Compared with acute wounds, persistent inflammation is one of the common characteristics of chronic wounds such as DUs [77]. Inflammatory factors and the infiltration of vascular and perivascular inflammatory cells result in chronic, systemic, low-grade inflammation. Due to risk factors such as hypoxia, oxidative stress and repeated infection, DFUs remain in the inflammatory stage of wound repair and cannot transition to the proliferative stage, creating chronic wounds that mainly present with great neutrophil and macrophage exudation and high levels of proinflammatory cytokines and proteases [78].

As the cornerstone cells of the immune system, neutrophils precede macrophages in the early stages of wound healing, clearing pathogens through phagocytosis and the secretion of antimicrobial substances and expressing a variety of cytokines and chemokines to lay the foundation for the subsequent healing process [79]. Diabetic neutrophils are defective in bactericidal functions, and Repine et al. observed that neutrophils isolated from diabetes patients killed *Staphylococcus aureus* much less effectively than the neutrophils from normal individuals, which was even more pronounced under poor glycemic control, so that despite the presence of large numbers of neutrophils in the ulcer, patients still have a high risk of infection. Interestingly, these defective neutrophils still produce extracellular ROS and proinflammatory cytokines [80, 81].

In acute wounds, the shift in macrophage phenotype from M1 to M2, with anti-inflammatory properties, is thought to be one of the important factors influencing the transition of wound healing from the inflammatory phase to the proliferative phase [82]. As described above, large amounts of accumulated AGEs in a wound can induce macrophages to polarize to the M1 phenotype, causing the wound phase to stay in the inflammatory stage and thus inhibiting the proliferation of fibroblasts, keratinocytes and endothelial cells [66, 83]. Macrophages also secrete a large number of proteases, among which MMP-9 can breakdown newly synthesized ECM and impede cell migration to inhibit wound repair [84, 85]. The disrupted ECM components can also act as chemotactic agents, attracting more inflammatory cells and causing a vicious cycle of inflammation.

Inflammation can lead to oxidative stress, and oxidative stress can aggravate inflammation, which is a difficult pair of conditions throughout the course of DFUs. Oxidative stress refers to a state of imbalance caused by the excessive production of ROS or decreased function of the antioxidant system of the body [86]. In diabetic wounds, inflammatory cells such as neutrophils and macrophages, as well as fibroblasts and endothelial cells, can produce large amounts of ROS. Excess ROS in the skin can activate cellular molecular signaling and interfere with angiogenesis and the secretion of numerous growth factors (GFs), which leads to poor wound healing [87]. Low levels of ROS are thought to be beneficial during wound healing [88]; conversely, a high level of ROS has a negative impact on fibroblasts and their functions [89]. Superoxide dismutase (ecSOD/SOD3) is the main antioxidant enzyme that eliminates ROS in the extracellular space. Compared with normal mice, SOD3 knockout mice showed higher ROS levels and lower fibroblast activity and TGF-β1 expression, which significantly delayed wound healing [90]. In addition, in dermal fibroblasts, ROS can increase MMP activity and accelerate the degradation of newly formed ECM by activating NF-κB and AP-1 [91]. Furthermore, keratin-forming cells, when stimulated by a highly oxidizing environment, can activate the EGFR-ERK pathway and release more IL-8 to recruit and activate neutrophils, creating a vicious cycle of inflammation-oxidative stress [92].

### Infection: a major cause of amputation

Infection is usually a consequence, not a cause, of diabetic DUs. Diabetic foot infections vary in severity, from simple cellulitis to limb- and life-threatening necrotizing fasciitis [93]. Diabetic foot infections can also encompass a wide variety of bacterial species, with most bacteria (*Staphylococci*, *Streptococci*, *Enterococci*, *Escherichia coli* and other gram-negative bacteria) [94]. More importantly, the presence of antibiotic-resistant strains is also common, especially methicillin-resistant S. aureus, which is present in 30% to 40% of cases [70]. In the late stage of DUs, due to the lack of foot sensations, skin damage and poor perfusion, bacteria rapidly penetrate deep into the fascia and cause fatal sepsis; thus, patients with DUs are often at risk of amputation. After amputation, trauma is amplified, and the patient's immunity is weaker, which further increases the risk of postoperative infection, creating a vicious cycle of "infection - amputation - infection". Therefore, it is particularly important to control infection as early as possible and to preserve the affected limb [16].

## Current treatments for DU

DU is a clinically common chronic difficult-to-heal wound with a prevalence of 8.1% in diabetic patients over 50 years of age. It is highly susceptible to combined infection, local circulatory disorders and nerve damage, which significantly increase amputation rates and mortality in patients if not controlled in a timely manner. In addition to basic treatments such as glycemic control and wound cleaning, new adjuvant treatments also play an important role in the treatment of DU. In recent years, many new adjuvant treatments for DU have been applied in clinical practice, such as vacuum sealing drainage (VSD) and new polymer dressings. It is worth noting that before treating DU, the character of the ulcer should first be assessed to distinguish whether it is a neurologic or ischemic ulcer or a neuroischemic ulcer. For ischemic ulcers, attention should be given to solving the ischemia of the lower extremities, while for neuropathic ulcers, the main focus is on foot decompression, with special attention given to the appropriateness of the patient's footwear.

The underlying cause of DU is the stimulation of local tissues by a long-term hyperglycemic environment, so effective glucose monitoring and glucose control protocols are prerequisites for the prevention and treatment of DU. Debridement is often the first step in treatment. The main purpose of debridement is to remove necrotic tissue, inhibit infection, and prepare the trauma environment for granulation tissue regeneration. In recent years, some new clinical debridement techniques have emerged, such as medical maggots and protein hydrolases. Maggot debridement is the use of medical-grade maggots to engulf necrotic tissue on the trauma surface. A randomized controlled trial including 267 patients with ulcers showed that compared to hydrogel treatment, larvae treatment significantly reduced the median time to debridement from 72 days to 14 days [95]. Enzymatic debridement occurs when exogenous protein hydrolases achieve debridement by selectively acting on necrotic tissue. However, known randomized controlled studies have a high risk of bias, numerous and varied outcomes, and an increasing risk of adverse events and therefore cannot be used as a basis for clinical practice [96].

When the debridement has reached a certain level, the VSD technique can be chosen to isolate the wound from the outside world by means of a negative pressure suction device. The negative pressure could effectively improve wound drainage, accelerate necrotic tissue lysis and granulation tissue proliferation, establish local blood supply of the wound, and provide a good environment for wound healing of diabetes ulcers [97]. In a Chinese clinical trial, 104 patients were divided into a conventional group and VSD group, and the conventional group was treated with systemic and local anti-infection therapy and local debridement and drainage; the VSD group was treated with the VSD technique in addition to conventional treatment. The experimental results indicated that compared with the conventional group, the VSD group had a significantly shorter healing time of the foot wound scar, an obviously lower number of dressing replacements, and a shorter average hospital stay, with a total clinical efficiency of 92.1%, which was higher than that of 79.2% in the conventional group. However, VSD treatment generally requires patients to be hospitalized and is not suitable for outpatients. Moreover, this treatment cannot replace debridement and requires control of local and systemic infection before use.

HBOT is considered an important adjunctive therapy to promote healing of DU wounds and requires that the patient be confined to a closed container and given 100% oxygen for breathing. It is controversial whether to apply HBOT to DU trauma. Evidence supports that HBOT can promote wound healing while also reducing the amputation rate to some extent. However, despite good experimental data in vitro and in many animal models, there is little effective clinical evidence of HBOT in the treatment of chronic wounds. In a double-blind randomized controlled study including 103 patients, HBOT was found not to reduce indications for amputation or promote wound healing compared to comprehensive wound care in patients with chronic diabetic foot ulcers [98]. Therefore, the evidence on HBOT as an adjunct to standard care for DFU remains inconclusive.

In the clinical treatment of diabetic foot ulcers, in addition to conventional medical treatment, the selection of external dressings is very important for the healing of DFUs. The role of dressings is to provide a moist environment, increase the local blood supply to the wound, promote the growth of granulation tissue, and shorten the healing time of the wound. Hydrogel dressings are one of the most widely studied new polymer dressings. There is clear evidence in animal experiments to support that hydrogels can shorten healing time and promote wound healing, but unfortunately, there are few laboratory translation and clinical studies on hydrogels. In a meta-analysis that included 446 subjects, a significant increase in hydrogel healing rates was found from an analysis of three studies comparing hydrogel dressings with basic wound contraction dressings [99]. However, this finding is not entirely certain due to the risk of bias in the original study. Therefore, it is particularly important to carry out high-quality and powerful clinical research on hydrogel therapy for DU.

## History of therapeutic materials for DU

At the end of the 18th century, Louis Pasteur started to use dry dressings to cover wounds to keep them dry and introduced the concept of asepsis and bacterial infection control, which pioneered dry healing. In 1962, Dr. Jorge Winter, a British zoologist, confirmed in animal experiments that wound healing was twice as fast in a wet environment as in a dry environment and proposed a wound dressing that kept the wound surface "sterile, moist and closed". He proposed the theory of wet wound healing to keep the wound "sterile, moist and closed", which broke through the theoretical limitations of Pasteur and Lister [100]. In 1960, Wichtlerle and Lim first developed and studied hydrogels based on hydroxyethyl methacrylate (PHEMA) as a biomaterial for contact lenses [101]. The temperature-dependent phase transition of pNIPAAm solutions in water was first reported by Heskins and Guillet in 1968 [102]. Three years later, Kopecek introduced ionic groups to the pHEMA backbone and designed the first pH-sensitive hydrogel [103]. Since then, hydrogels have been used in a wide variety of biomedical applications, such as tissue engineering, drug delivery systems, contact lenses, and wound dressings.

In 1998, J L Jensen et al. conducted a study comparing the healing efficacy of Carrasyn® hydrogel and saline gauze for DU. The study showed that the incidence of complete wound closure was significantly higher in the Carrasyn® gel treatment group and that the healing time was also significantly shorter [104]. However, due to the lack of vascularity and nutrition in diabetic ulcer wounds, it is clear that dressing therapy alone is not sufficient. Therefore, a large number of bioactive substances (e.g., growth factors, peptides, mRNA, etc.) have been added to hydrogels for the treatment of DU. Kiyohaya Obara et al. in 2003 prepared a photocrosslinked chitosan hydrogel containing fibroblast growth factor-2 for stimulating wound healing in healing-impaired db/db mice [105]. However, the problems associated with a high degradation rate, low bioavailability, and high cost are also troubling.

**Fig. 5:**
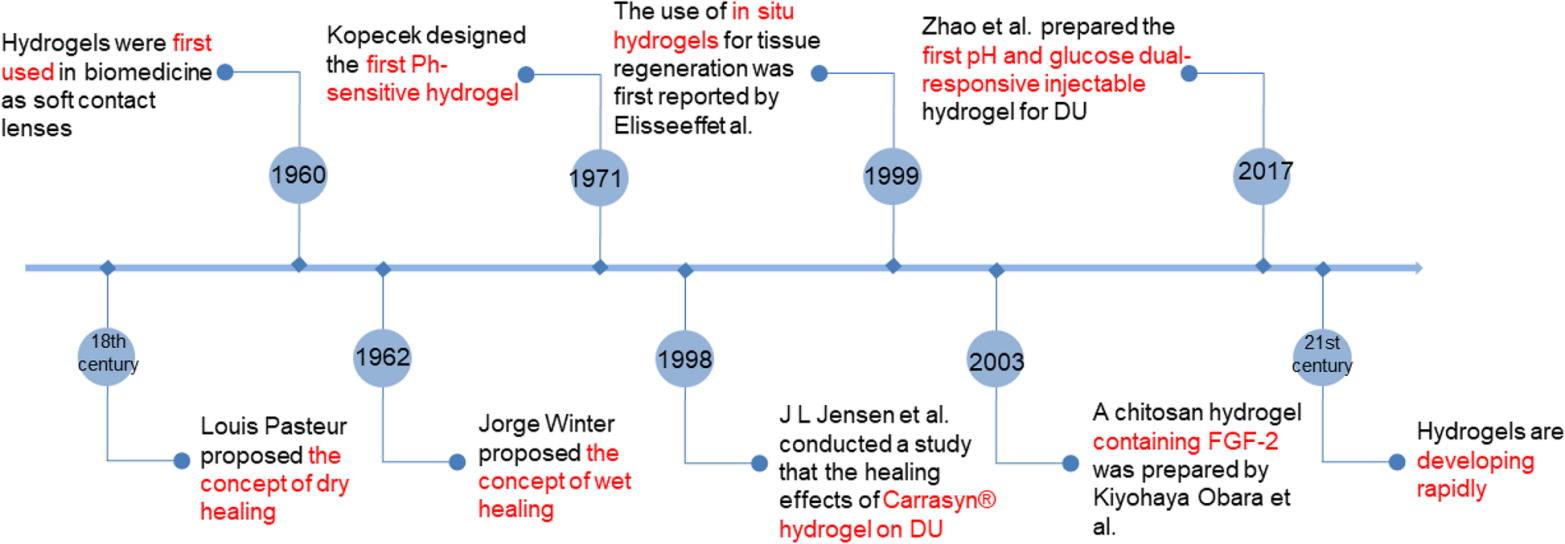
A timeline of materials for treating DU

Notably, because the shape of the hydrogel is usually determined in vitro, this creates a gap between the wound and the dressing that does not maximize healing. Moreover, for deeper diabetic ulcers, surgical implantation of the hydrogel is also needed, which can be physically and mentally taxing for the patient. Therefore, the concept of injectable in situ hydrogels was introduced, and the use of in situ hydrogels for tissue regeneration was first reported by Elisseeff et al. in 1999 [106]. The in situ formed gel will be formed in a polymer solution; after injection, the polymer solution undergoes physical and chemical cross-linking to form the gel and provide the proper connection between the gel and the host tissue. In recent years, a variety of injectable hydrogels have been developed for the treatment of DU. In addition, the development of responsive hydrogels has boomed with the advancement of technology and the needs of DU patients. Zhao et al. used cross-linking of benzene boron-modified chitosan, polyvinyl alcohol and benzaldehyde-capped polyethylene glycol to prepare the first pH and glucose dual-responsive injectable hydrogels for the treatment of DU [107]. From the initial exploration to the present, material scientists and surgeons have been dedicated to exploring the advanced properties of hydrogel-based materials with a view to their possible role in the treatment of DU (Fig. 5).

## Advanced polymer hydrogels for DUs

Hydrogels are a new type of functional polymer material that are widely used in the biomedical field because of their high water content, fast swelling rate and good biocompatibility [108]. According to the material used for their synthesis, hydrogels can be classified as natural hydrogels and synthetic hydrogels [109], with natural hydrogels being divided according to substrate: chitosan (CS), hyaluronic acid (HA), alginate, gelatin, cellulose, etc. Hydrogels have a three-dimensional network structure formed by physical or chemical crosslinking. Physical crosslinking mainly occurs through intermolecular forces, such as electrostatic/ionic, hydrophobic, and hydrogen bonding interactions. Chemical crosslinking results from covalent bonds that form through chemical reactions, and since covalent interactions are much stronger than the noncovalent interactions, chemically crosslinked hydrogels have better mechanical stability [110–112]. Compared with traditional wound dressings, hydrogels keep wounds moist and continuously absorb exudate, and some hydrogels prepared from chitosan and alginate can degrade over time to avoid secondary damage during dressing changes [24]. More importantly, scientists can endow hydrogel dressings with a variety of excellent properties through structural design and functional integration, which play important roles in the DU healing process. However, the mechanical stability of hydrogels in the swollen state is poor and does not meet the needs of treatment [113]. Moreover, some hydrogels need to be covered with a secondary dressing to prevent dehydration [113]. In this section, we introduce different advanced polymer hydrogels and describe their inherent characteristics and applications in the field of DUs in detail.

### Chitosan-based hydrogels

Chitosan is a partially deacetylated product of the natural polysaccharide chitin, which consists of glucosamine and N-acetylglucosamine units [114]. Chitosan polymers have many active functional groups, such as amino and hydroxyl groups [114, 115]. Therefore, chitosan has properties to similar antibiotics. As the mode of action of chitosan is affected by different factors, it is difficult to clarify the specific mechanism behind its antimicrobial activity.

At present, the following opinions on chitosan exist in academia. 1. Under physiological conditions, chitosan and its derivatives are polycations that interact with negatively charged microbial cell surface residues through electrostatic interactions, causing cell wall peptidoglycan hydrolysis and changing cell membrane permeability. Moreover, due to the polycationic nature of chitosan, it can accumulate on the surface of microbial cells, stopping the pathway of bacterial substance exchange and causing microbial death. 2. The amino and hydroxyl groups on chitosan molecules chelate with trace metal ions (such as calcium and zinc ions), affecting the normal metabolism of microbial cells. 3. Chitosan can cross microbial cell walls and cell membranes, enter the interior of bacteria, and bind to nucleic acids, thus interfering with normal cellular gene transcription and protein expression. Chitosan has broad-spectrum inhibitory activities and a high rate of inactivation against both gram-positive and gram-negative bacteria but low cytotoxicity to mammals, which is the prerequisite for its wide application in the biomedical field [116–120]. In addition to its excellent antibacterial properties, chitosan has good biocompatibility. Since chitosan itself carries a positive charge, it could combine with negatively charged macromolecules on the cell surface for biocompatible electrostatic binding. Some studies have shown that chitosan that had been implanted in animals could be completely degraded within 14 days. Chitosan also mimics the normal ECM environment, stimulates the growth of granulation tissue, and induces collagen and elastin secretion by fibroblasts, thus promoting cell proliferation and tissue repair [121–123]. Ana Aglahe Escárcega-Galaz et al. applied 2% chitosan gel and chitosan film to the wound areas of eight diabetic patients with skin ulcers and showed that the use of chitosan reduced the growth of gram-negative and gram-positive bacteria, in accordance with the laboratory results. More excitingly, while only one patient experienced complete healing of the ulcer, all patients showed significant improvement in the initial wound with formation of granulation tissue and healthy skin coverage, demonstrating the great potential of chitosan applications in DU [124].

**Fig. 6:**
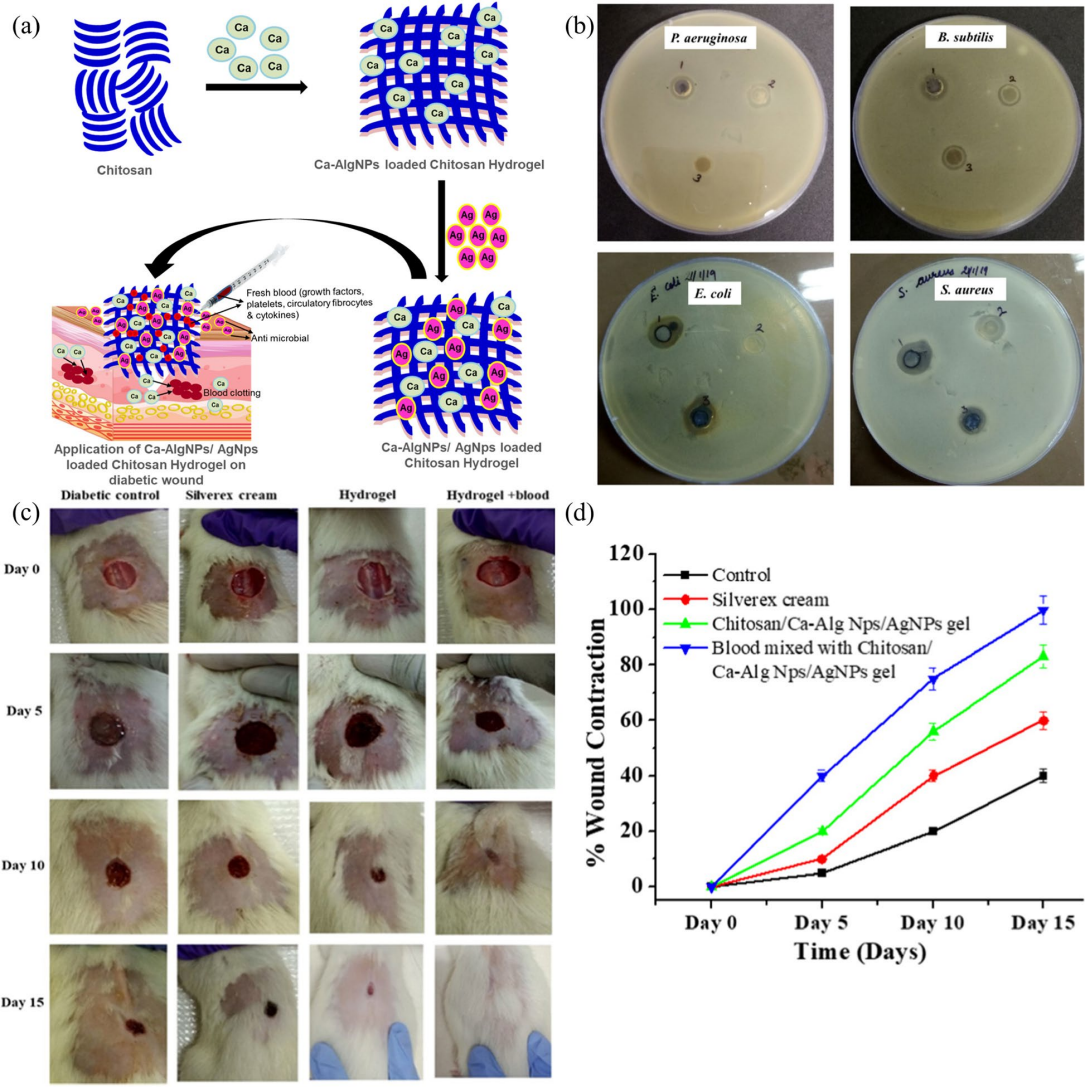
Blood mixed Chitosan/Ca-AlgNps/AgNPs hydrogel for DU (**a**) Fabrication design of Ca-AlgNps and AgNps loaded hydrogel (**b**) Antimicrobial behavior of AgNPs (1), Chitosan/Ca-AlgNPs (2), and Chitosan/Ca-AlgNps/AgNPs (3) against P. aeruginosa, B. subtilis, E. coli and S. aureus. **c** Wound contraction in different wound dressing groups at day 0, day 5, day 10 and day 15 postexcision injury. **d** Wound reduction area (%) in different wound healing groups. Reproduced from [125] with permission from Elsevier Copyright 2021

Because of its excellent antibacterial properties and independent degradability and good biocompatibility, many scientists have combined the active functional groups of chitosan with metal ions, nanoparticles, and bioactive glasses to develop chitosan hydrogels with different functions for the treatment of patients with DUs. Choudhary et al. developed a novel biodegradable and self-absorbable hydrogel dressing using chitosan as a matrix, Ca-AlgNps as a hemostatic agent and AgNps as an antibacterial agent (Fig. 6 (a)). The prepared chitosan/Ca-AlgNps/AgNP hydrogel exhibited broad-spectrum antibacterial properties against both gram-negative bacteria (Escherichia coli, Pseudomonas aeruginosa) and gram-positive bacteria (Bacillus subtilis, Staphylococcus aureus) (Fig. 6 (b)). The combination of chitosan with silver nanoparticles is not new. It is worth mentioning that the researchers proposed replacing the growth factors necessary for wound repair with fresh blood, which has not been seen in previous studies. Moreover, comparing Chitosan/Ca-AlgNps/AgNPs, blood-mixed Chitosan/Ca-AlgNps/AgNPs demonstrated significant wound healing effects (Fig. 6 (c)). The chitosan/Ca-AlgNps/AgNPs- and blood mixed with chitosan/Ca-AlgNps/AgNPs-treated groups exhibited wound contraction of 21.5 ± 1.12% and 40.25 ± 2.41% on the 5th day, 56 ± 1.81% and 75.2 ± 4.12% on the 10th day, and 83.52 ± 4.38% and 99.76 ± 1.98% on the 15th day, respectively (Fig. 6 (d)). This demonstrates the potential of blood-mixed novel hydrogels as growth factor substitutes and tissue repair for chronic diabetic wounds [125]. However, the following issues also require attention. Blood should be checked microbiologically before topical application to the wound to ensure experimental safety, which is not reflected in the text. Second, despite topical application, rejection may still occur between the foreign blood and the human body environment, and some immune-related indicators should be tested to clarify the severity of rejection. Moreover, as time passes, the growth factors and active ingredients within fresh blood may gradually decrease or become inactivated, and this issue is the biggest obstacle to the clinical practice of blood-mixed hydrogels.

Researchers have found that hydrogels prepared by mixing chitosan with synthetic polymer materials often have excellent comprehensive properties, which can greatly compensate for the shortcomings of chitosan hydrogels, such as their insufficient mechanical properties and poor stability. Wang et al. prepared PVA/chitosan hydrogels and introduced traditional Tibetan eighteen flavor dangshen pills (TEPs) for the treatment of DU. The researchers prepared PVA/CS hydrogels containing 0%, 5%, 10% and 20 wt % TEP for physical property testing. First, the storage modulus (G′) and loss modulus (G″) were tested. The experimental results showed that the G′ and G″ of the four groups of hydrogels were relatively stable between 20℃ and 40℃, and the G′ of the four groups of hydrogels were always larger than G″, which indicated that these gels were always in the gel state. In addition, the researchers also tested the mechanical properties of the hydrogels, and the tensile test showed that with increasing TEP content, all four hydrogels had good tensile ability (88.7%-281.2%), which was better than the human skin ductility (60-75%). In addition, since TEP as solid particles replaced the original soft hydrogel, the strain increased from approximately 0.15 MPa to 1.18 MPa when the TEP mass ratio was increased from 0% to 5%, 10% and 20% with a deformation of approximately 90%, suggesting that the hydrogel was able to withstand the compressive strain. However, unfortunately, the swelling rate of the prepared hydrogel can be as high as 313.5%, which is not conducive to maintaining the original shape of the wound and keeping it moist. Additionally, of note is the introduction of the traditional Tibetan medicine TEP, which consists of 18 traditional Tibetan medicines, including Tibetan ginseng, cassia seeds and alpine coriander, and has anti-inflammatory and analgesic properties as well as pro-healing properties [126]. Also, Masood et al. reported the development of a silver nanoparticle (AgNP)-impregnated chitosan-poly ethylene glycol (PEG) hydrogel and characterized it using ultraviolet (UV)–visible spectrophotometry, Fourier transform infrared (FT-IR) spectroscopy and scanning electron microscopy (SEM). The results showed that the silver nanoparticle-impregnated hydrogel had higher porosity and swelling and a greater water vapor conversion ratio (WVTR), as well as superior antibacterial and antioxidant properties and an enhanced wound healing ability in diabetic rabbits [127].

The presence of chitosan-based dressings such as KytoCel, Axiostat®, KA01 chitosan wound dressing, ChitoClear®, Celox™, ChitoHeal, etc., in the market indicates the great potential of chitosan-based hydrogels for wound management. Chitosan-based hydrogels cross-linked with synthetic polymers show interesting mechanical properties for wound management, including good elasticity, flexibility, compressive stress, Young's modulus and tensile stress. Even so, there are still some obstacles to overcome. Because the raw materials for chitosan are mostly from natural sources, due attention should be given to the potential risk of batch-to-batch variation. In addition, there are many ways to prepare hydrogels with desirable properties based on laboratory studies, and a large number of functions can be imparted to hydrogels by a variety of methods. However, considering industrial production, how to reduce manufacturing costs and simplify the production process with minimal contamination also needs attention. Table 1 shows the major applications of chitosan-based hydrogels in DFUs over the past five years.

**Table 1:** Chitosan-based hydrogels

System	Substance	Mechanism	Signal pathway	Cell line	Animal	Wound size	Characteristic	Ref.
Chitosan/heparin/poly (γ-glutamic acid)	Superoxide dismutase	Re-epithelialization and collagen deposition (+)	NA	3T3 fibroblasts.	STZ-induced diabetic rat model	Two,1cm	Good rheological, swelling, biocompatibility, antioxidant properties	[128]
Chitosan and PVA	Zn2+, polypyrrole	NA	NA	Fibroblasts	STZ-induced diabetic rat model	Four ,1 cm	Excellent stretch, self-healing, biocompatibility, conductivity, antibacterial activity	[129]
Chitosan/polyurethane/PLGA	BMMNCs	Neovascularization (+); wounds size, inflammation (-)	NA	BMMNCs	STZ-induced diabetic rat model	One,0.6cm	Low cytotoxicity, promote healing	[130]
Quaternized chitosan, benzaldehyde-terminated F108 (F108-CHO)	CORM-401, insulin	Anti-inflammatory, Antibacterial, antioxidative stress (+)	ATP synthesis, activated macrophages proliferation (-); scavenging ROS, the expression of HO-1, the polarization of M1 to M2(+); the rupture of bacterial membranes, mitochondrial dysfunction	L929 cells and HUVECs	STZ-induced diabetic rat model	One,0.8cm	Good tissue adhesion, injectability, self-healing, controllable insulin release ability	[131]
Chitosan and decellularized dermal matrix	Carbon nanodots, hAMSC	Angiogenesis, collagen deposition, wound closure, re-epithelialization, formation of DEJ (+)	ColI(+), ColIII, KRT(-)	Human foreskin fibroblasts	STZ-induced diabetic rat model	One,2cm	Proangiogenic potential, ROS scavenging, hemocompatibility, and moderate antimicrobial activity	[132]
Chitosan/PVA	Perfluorocarbon emulsions, EGF-loaded chitosan nanoparticles, and polyhexamethylene biguanide	Re-epithelization, collagen deposition and maturation (+); inflammatory (-)	IL-8(-)	Human keratinocytes (KERTr cells; ATCC® CRL-2309™)	STZ-induced diabetic rat model	Two,0.8 cm	Oxygen delivery, antibacterial, anti-inflammation, and promotive cell growth	[133]
Chitosan	Mixture of flavonoids isolated from the leaves of *Passiflora edulis*	Antioxidant defense system (+)	NA	Fibroblasts (L929)	Alloxan-treated rats	One,1cm	Anti-inflammatory, antioxidant	[134]
Quaternized chitosan, Tannic acid	/	Collagen deposition, no scar formation, skin regeneration (+)	NA	Rat skin fibroblast-like (RS1) cells	STZ-induced diabetic rat model	One,1cm (Not mentioned in the text, estimated by pictures)	Good injectability and self-healing, cytocompatibility, hemostatic capability and biodegradability, radical scavenging activity	[135]
Fluorinated Meth acrylamide Chitosan	/	Collagen synthesis, neovascularization (+)	NA	NA	The (db/db) homozygote mice	NA,0.8cm	No bioaccumulation, oxygenating	[136]
Ulvan dialdehyde/chitosan/dopamine	Ag NPs, hUC-MSCs	Cell proliferation and migration (+)	Capase3-dependent BCL2 expression, the activation of PCNA and AKT(+)	NIH/3T3 cells	STZ-induced diabetic rat model	One,1cm	Adequate mechanical properties, swelling capability, adhesiveness, antioxidant, antibacterial ability	[137]
Chitosan	PRP, silk fibroin	Collagen deposition, angiogenesis, and nerve repair (+)	CD34, NF-200(+)	L929 cells, HDFs, HUVECs, HUMSCs	STZ-induced diabetic rat model	One,1cm	Injectable, self-healing, biodegradable	[138]
CS-DA-LAG, PEG-PBA-BA	Metformin, polydopamine-coated reduced graphene oxide	Inflammation (-), angiogenesis (+)	IL-6(-); α-SMA, CD31(+)	HUVECs	Type 2 diabetes rat model	One,0.5cm	pH/glucose dual-responsive, adhesion-enhanced, self-healing, easy-removable, antibacterial, antioxidant, conductive, hemostasis	[139]
PVA/Chitosan	The tibetan eighteen flavor dangshen pills	Collagen deposition (+); Pro-inflammatory cytokines (-)	TNF-α, IL-6, IL-1β (-)	L929 cells, HUVECs	STZ-induced diabetic rat model	Four,1cm	Good rheology, suitable swelling and degradation behavior, excellent biocompatibility and antibacterial activity	[126]
Chitosan	Calcium alginate nanoparticles, AgNPs, fresh blood	Collagen bundle (+)	NA	NA	STZ-induced diabetic rat model	One,1.2cm	Antimicrobial, hemostatic, self-healing, scar free	[125]
Chitosan-PEG	AgNPs	NA	NA	NA	Alloxan-induced diabetic rabbits’ model	Two,2cm	Antioxidant, antibacterial	[127]
Methacrylated chitosan	P. granatum peel crude extract, AgNPs, ethyl acetate fraction	NA	TGF-β1, NF-κB (+)	Human normal cell line (HFB4)	Diabetic rat model	one,1cm	Good mechanical characterization, promote healing	[140]
Benzaldehyde-terminated 4-arm PEG/carboxymethyl chitosan	bFGF	Epithelialization, collagen, hair follicles, neovascularization (+)	Ki67, CD31, CD34 expression (+)	L929 cells	STZ-induced diabetic rat model	One,1cm	Excellent wet-tissue adhesion, self-mending, and antibacterial, biocompatibility and fast hemostasis capacity	[141]
Quaternized chitosan, ε-poly-L-lysine grafted graphene quantum dots, benzaldehyde-terminated four-arm poly (ethylene glycol)	/	The migration and proliferation of fibroblast cell, inactivation of bacteria (+); bacterial membrane (-)	NA	NIH3T3 cells	STZ-induced diabetic rat model	One,1cm	pH sensitivity, self-healing, hemostatic, and biocompatible	[142]
Collagen, Chitosan	Silver nanoparticles	Collagen deposition, hair follicle repair, sebaceous glands formation (+)	VEGF, TGF-b1, IL-1b, and TIMP1 expression (+)	HUVECs	alloxan-induced diabetic rat model	One,0.5cm	Good mechanical properties and biological activity	[143]
Chitosan	Puerarin	Angiogenesis (+); inflammation (-)	MiR-29ab1 mediated inflammatory axis (-)	Mouse mononuclear macrophage leukemia cells (RAW264.7)	STZ-induced diabetic rat model	One,1cm	Anti-inflammatory, promote healing	[144]
Sulfated chitosan	Collagen type I	Re-epithelialization, collagen deposition, neovascularization (+)	IL-4, TGF-β1(+); IL-6(-)	L929 fibroblasts	STZ-induced diabetic rat model	Two, NA	Improving inflammatory microenvironment, regulate macrophage behaviors	[145]
PVA, chitosan, phenylboric acid	Insulin, gelatin microspheres containing celecoxib	Angiogenesis, cell proliferation and migration, hair follicle regeneration (+)	IL-10, VEGF (+); TNF-α, MMP9, AGEs (-)	L929 cells, RAW 264.7 macrophages	STZ-induced diabetic rat model	Two ,0.8cm	Glucose and MMP-9 dual-response, temperature-sensitive, Shape self-adaptive, excellent biocompatibility	[146]
Chitosan	Ag^+^, Cu^2+^	cell migration, angiogenesis (+)	IL-10, α-SMA, VEGF, CD31 (+)	HUVECs and mouse fibroblast cells (L929)	STZ-induced diabetic rat model	NA,0.8cm	Injectable, self-healing, and biodegradable, antibacterial	[147]
Chitosan HCl, κ- carrageenan and PVA	Cefotaxime	Angiogenesis, collagen deposition, epidermis regeneration (+)	NA	NIH3T3 fibroblast cells	STZ-induced diabetic rat model	NA,1.5 cm (burn)	Excellent bacterial barrier property, oxygen and water transmission rate, flexibility, mechanical properties	[148]
Pluronic F127 and chitosan	Ce@LTA-NPs	The formation of granulation tissue, re-epithelialization, collagen remodeling (+)	NA	HUVECs,murine macrophage RAW264.7	STZ-induced diabetic rat model	One,2cm	ROS scavenging, clear inflammatory factors, good porosity, stability and biocompatibility	[149]

“(+)” represents upregulated or promoted

“(-)” represents downregulated or suppressed

### HA-based hydrogels

HA is a polysaccharide that was isolated by Meyer in 1934 from the vitreous fluid of bovine eyes [150]. Its chemical structure consists of repeating disaccharide units of D-glucuronide and N-acetyl-D-glucosamine linked by alternating β-1,4 and β-1,3 glycosidic bonds. HA is widely distributed in the ECM of epithelial, soft connective and neural tissues of vertebrates [151]. Hyaluronan is mainly recognized by receptor differentiation cluster 44 (CD44), HA-mediated motor receptor (RHAMM), HA endocytosis receptor (HARE) and lymphatic vessel endothelial cell receptor 1 (LYVE-1), which activate intracellular signaling pathways and participate in the process of tissue repair and the inflammatory response [152].

**Fig. 7:**
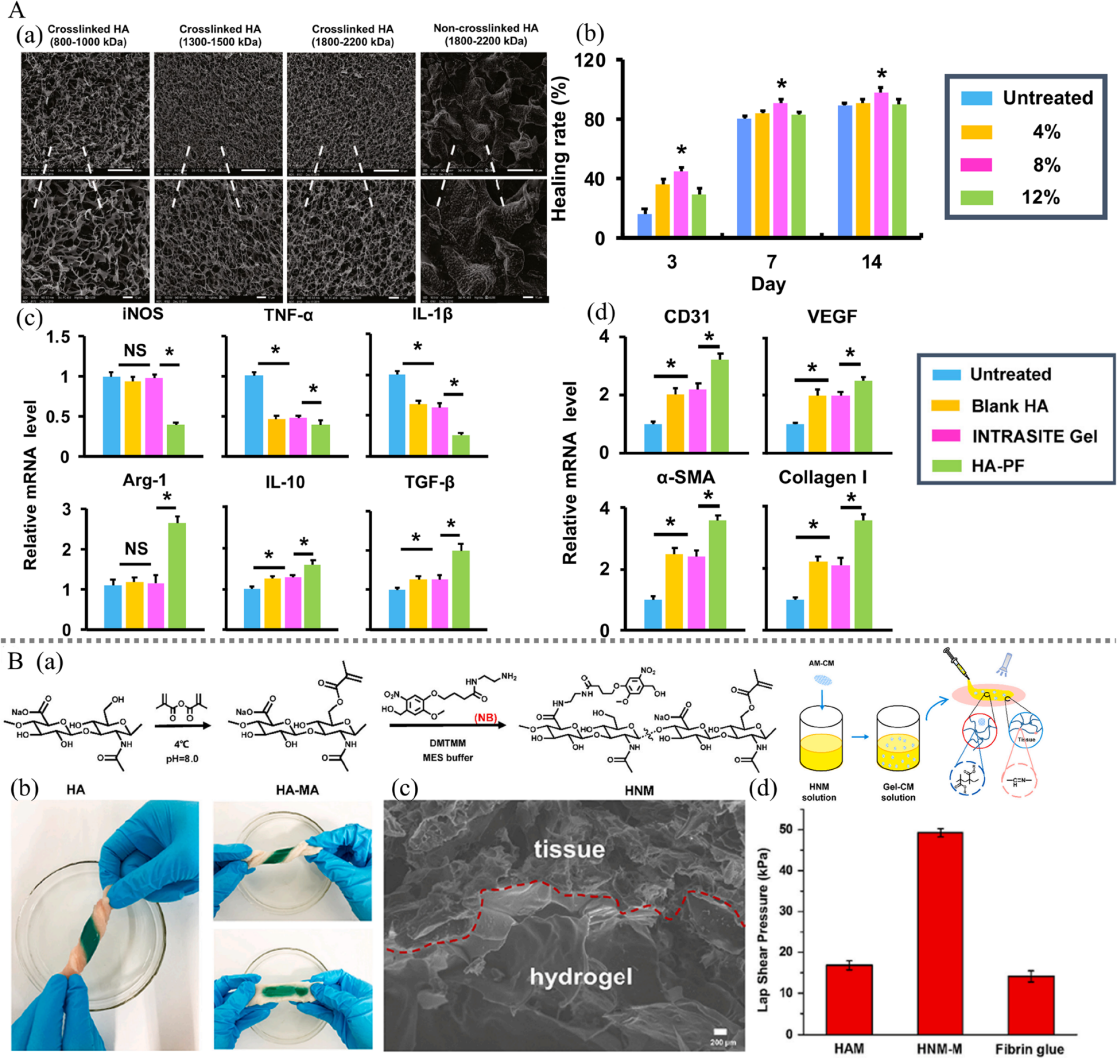
A Modulation of macrophages by a paeoniflorin-loaded hyaluronic acid-based hydrogel (HA-PF) promotes diabetic wound healing. **a** SEM image of cross-linked HA with different molecular weights (Mw). **b** The healing rate (%) of mice with full-thickness wounds following treatment with crosslinked hydrogels (Mw ¼ 1,800–2,200 kDa) at concentrations of 4%, 8% and 12%. **c** The mRNA expression of cytokines in the wound following different treatments. **d** The mRNA expression of CD31, VEGF, α-SMA and type I collagen in the wound following different treatments. **B** HA-based hydrogel grafted with methacrylic anhydride and N-(2-aminoethyl)-4-[4-(hydroxymethyl)-2-methoxy-5-nitrophenoxy]-butanamide (NB) groups to encapsulate lyophilized amnion-derived conditioned medium (AM-CM). **a** Schematic illustration of the preparation process of the HNM hydrogel encapsulated with freeze-dried AM-CM. **b** Photographs of the in situ formation of the HNM-M hydrogel (200 μL, 2 wt%) on pork skin after light irradiation. No detachment or breakage between the pork skin and hydrogel was observed after being stretched or twisted. **c** SEM image of tissue section of in situ formed hydrogel in pork skin. **d** Scheme of the lap-shear adhesion test to determine the hydrogel-tissue binding strength and quantification of the adhesive strength of the HNM-M hydrogel. Reproduced from [156, 157] with permission from Elsevier Copyright 2021, 2022

CD44 is the main receptor of HA, and when HA binds to CD44, it can promote angiogenesis through the MAPK/ERK signaling pathway, resulting in ERK1/2 activation and an increase in the migration of endothelial cells [153]. In addition, HA is also involved in the immune response during wound repair. High molecular weight hyaluronic acid (HHA) can promote the production of IL-2, IL-10 and TGF-β by upregulating Foxp3 expression in Tregs, inhibiting the inflammatory response and promoting wound repair [154]. In recent years, researchers have found that HA can improve wound healing by modulating macrophages [151, 155]. Macrophages can be activated to polarize into the proinflammatory M1 phenotype or the anti-inflammatory M2 phenotype. More macrophages can be polarized to the M2 phenotype by modulating local immunity, which has been explored as a new therapeutic strategy to promote diabetic wound healing. Yang et al. developed a high molecular weight hyaluronic acid (HA)-based hydrogel loaded with paeoniflorin (PF), which successfully achieved a PF-mediated transition of macrophages from a pro-inflammatory M1 phenotype to an anti-inflammatory M2 phenotype. The SEM results showed that HAs cross-linked by ADH exhibited a porous three-dimensional morphology, indicating the ability to load PF (Fig. 7 A (a)). It is known from previous experiments that the mobility of hydrogels decreases with increasing concentration. In this study, cross-linked hydrogels at concentrations of 4%, 8%, and 12% were applied to full-length wounds in mice. The results showed that the 8% hydrogel accelerated wound closure compared to the 4% and 12% hydrogels (Fig. 7 A (b)). This may be because the 8% hydrogel reached a viscosity that facilitated topical administration. The results of animal experiments showed that HA-PF could promote macrophage polarization from M1 to M2 in late inflammation, downregulate iNOS, TNF-α, IL-1β, and Arg-1, upregulate IL10 and TGF-β, and thus improve inflammation (Fig. 7 A (c)). In addition, HA-PF significantly increased the expression of CD31, VEGF, α-SMA and type I collagen at day 14 after injury (Fig. 7 A (d)). This suggests a role for HA-PF in promoting angiogenesis, re-epithelialization and collagen production. Furthermore, it is noteworthy that HA-PF was able to significantly improve healing relative to INTRASITE gel (a commercial hydrogel wound dressing) (Fig. 7 A (c-d)). This offers great promise for the clinical translation of HA-PF in diabetic wound healing [156].

It is well known that both strong adhesion and large deformability are critical for wound dressings, as wounds are often in a highly dynamic environment. Zhang et al. engineered a hyaluronic acid-based hydrogel grafted with methacrylic anhydride (MA) and N-(2-aminoethyl)-4-[4-(hydroxymethyl)-2-methoxy-5-nitrophenoxy]-butana-mide (NB) groups to encapsulate lyophilized amnion-derived conditioned medium (AM-CM) (Fig. 7 B (a)). The investigators evaluated the adhesion properties of the hydrogels in vitro by a twisting test. This was visualized by grafting malachite green dye onto the hydrogel. No breakage or separation of the adhesive hydrogel from the pork skin was observed after the pork skin surface was gelatinized in situ and stretched or twisted (Fig. 7 B (b)). They further observed the interface between the pork skin and the hydrogel by SEM and found a seamless interface with tight contact (Fig. 7 A (c)). To quantify the adhesive strength, they performed lap shear tests, which reflect the ability of the hydrogel to adhere to the tissue when subjected to shear forces. The lap shear strength of the prepared hydrogel was 49.3 ± 1.0 kPa, which was three times stronger than that of the hydrogel without the NB group and stronger than that of fibrin glue (14.1 ± 1.3 kPa, an adhesive widely used in clinical practice) (Fig. 7 B (d)). Tensile tests showed that the prepared hydrogels remained highly extensible, maintaining 2.3 times the initial length before debonding from the tissue surface [157]. In addition, the loaded AM-CM was surprising. Early on, in a pilot study using cryopreserved amnion to promote wound healing after dental implant surgery, the amnion demonstrated an excellent ability to support epithelial growth and promote cell migration and adhesion. In this study, it was also validated in in vitro and in vivo experiments that the prepared hydrogel could promote diabetic wound healing by modulating macrophage polarization and promoting angiogenesis.

The commonly used HA-based hydrogels on the market are Restore ® Hydrogels and Regenecare ® HA. As a natural polysaccharide produced during the proliferation period of wound healing, HA also has advantages that other hydrogels do not have. The antigenicity of HA is much lower than that of other hydrogels [151, 153]. Moreover, HA is also recognized as one of the most moisturizing polysaccharides, which helps to lock in moisture and promote skin repair [153]. Table 2 shows the major HA-based hydrogels used in treating DUs over the last five years. However, despite the many advantages of HA, unmodified HA is rapidly degraded by hyaluronidase in body fluids, resulting in a short half-life in vivo [158]. Therefore, HA must be chemically modified before it can be used in tissue engineering and drug delivery. Sílvia Pérez-Rafael et al. developed multifunctional, injectable hydrogels composed of thiolated hyaluronic acid (HA-SH) and bioactive silver-lignin nanoparticles (Ag@Lig NPs). In vivo experiments showed that 15 days of nanohydrogel treatment completely restored wounds in both nondiabetic and diabetic mice, demonstrating its great potential for chronic wound management [159].

**Table 2:** HA-based hydrogel

System	Substance	Mechanism	Signal pathway	Cell line	Animal	Wound size	Characteristic	Ref.
Thiolated HA	Silver-lignin nanoparticles (Ag@Lig NPs)	Angiogenesis (+)	MPO, MMPs, ROS (-)	Keratinocytes (HaCaT cell line)	STZ-induced diabetic rat model	Two,0.2cm	Shear- thinning and self-healing, antibacterial and antioxidant, tunable physico-mechanical properties and degradation rate	[159]
HA grafted with methacrylic anhydride and N-(2-aminoethyl)-4-[4-(hydroxymethyl)-2-methoxy-5-nitrophenoxy]-butana-mide (NB) groups	Amnion-derived conditioned medium (AM-CM)	Mcrophage polarization, promoting angiogenesis (+)	NA	HUVECs, macrophages from the bone marrow of C57BL/6 mice, fibroblasts	The db/db mice	Two, NA	Excellent mechanical properties, elasticity, biocompatibility, tissue adhesion	[157]
HA, Pullulan, Pluronic® F127	Curcumin	Reepithelization, angiogenesis, collagen deposition (+)	NA	3T3-L1 fibroblasts cells	STZ-induced diabetic rat model	NA, 2 cm	Injectable, good rheological and mechanical properties, sustained drug release	[160]
HAMA, PBA	Catechin	Antioxidant enzyme activity, collagen deposition, angiogenesis (+); inflammatory responses, ROS levels (-)	VEGF, CD31, IL-10 expression (+); IL-6 (-)	Fibroblasts	STZ-induced diabetic rat model	One, 1cm	Glucose-responsive, antioxidant, self-healing	[161]
Oxidized dextran, antimicrobial peptide-modified HA	PRP	Collagen deposition, angiogenesis (+); inflammation (-)	TGF-β1, VEGF (+); TNF-α, IL-1β and IL-6(-)	L929 fibroblast cells	The db/db mice	Two,0.8 cm	Wide-spectrum antibacterial, good biocompatibility, stable rheological properties, biodegradability,	[162]
HA	Paeoniflorin	Angiogenesis, re-epithelialization, collagen deposition (+); inflammation (-)	The transition of macrophages from M1 to M2(+)	L929 cells (mouse fibroblasts)	STZ-induced diabetic rat model	Two full-thickness wounds (~0.3 cm2) per animal	Promote healing	[156]
HA, PBA, polyethylene glycol diacrylates	Myricetin	Angiogenesis, tissue remodeling (+); inflammatory response (-)	IL-10, VEGF, CD 31(+); IL-6(-)	NIH 3T3 cells	STZ-induced diabetic rat model	One, 0.8 cm	Glucose-responsive drug release	[163]
HA, heparin	Micellization curcumin	Re-epithelialization and collagen deposition (+)	The transition of macrophages from M1 to M2(+)	L929 cells	STZ-induced diabetic rat model	Two, 0.8cm	Good viscoelasticity and self-healing properties, excellent ROS scavenging ability and anti-inflammatory	[164]
HA, collagen	Gelatin encapsulated metformin microspheres	Collagen deposition (+); inflammation (-)	The transition of macrophages from M1 to M2(+)	Mouse NIH-3T3 cells and RAW 264.7 cells	High sugar and high-fat feed rats	Two ,1cm	pH-responsive, anti- inflammatory	[165]
Oxidized HA, (PBAimBF4)	NA	NA	VEGF (+); TNF-α (-)	L929 cells	Type I diabetes rat model	One,0.8cm	Injectable, satisfactory mechanical properties and flexibility, suitable conductivity and biocompatibility	[166]
HHA, PVA	M2 phenotype macrophages (MΦ2), Cu2+	Inflammation (-)	the transition of macrophages from M1 to M2(+)	Mouse fibroblast cell (L929), HUVECs, and mouse macrophage cells (Raw 264.7)	STZ-induced diabetic rat model	A 7mm thick wound on the back	Promote healing	[167]

“(+)” represents upregulated or promoted

“(-)” represents downregulated or suppressed

### Alginate-based hydrogels

Alginate is a natural anionic polymer derived mainly from brown algae found in the ocean. Alginate is a copolymer with L-gulo-glucuronide (G) and D-mannuronic acid (M) structural units with either continuous G chain segments (GGGG) and M chain segments (MMMM) or alternating MGMG chain segments [168, 169]. It is currently believed that only the G unit is involved in the multivalent ion-induced crosslinking of alginate and that a high G content can improve the mechanical strength of a hydrogel. Compared to other hydrogels used in DUs, alginate has unique advantages. 1. High hygroscopicity. Alginate dressings are extremely hydrophilic and can be used for highly exuding wounds [170]. 2. Gel obstruction. Alginate hydrogels show great expansion after contacting exudate, resulting in a reduction in the fine pore structure between the gels and a consequent slowing of the rate of fluid dispersion, allowing the wound exudate to disperse and greatly reducing the effect of infestation on healthy tissue [171]. 3. Most importantly, the alginate hydrogel does not adhere to the wounded tissue or cause secondary damage after removal. In a randomized controlled trial that included 18 patients with refractory wounds, investigators found increased ESC proliferation and differentiation in patients treated with a combination of alginate dressing and mEGF compared to those treated with mEGF alone, demonstrating the great potential of alginate in the field of wound management. Currently commercially available alginate saline gels are Purilon^®^ Gel and Nu-Gel^™^ etc. Table 3 shows the last five years of laboratory alginate hydrogel.

**Table 3:** Alginate-based hydrogel

System	Substance	Mechanism	Signal pathway	Cell line	Animal	Wound size	Characteristic	Ref.
Calcium alginate	Protamine nanoparticles, protamine NPs/HAO Ca-alginate hydrogel	Granulation tissue formation, angiogenesis, wound contraction, and re-epithelialization (+); inflammation (-)	VEGF (+)	HUVECs, Human foreskin fibroblasts	STZ-induced diabetic rat model	One,2cm	Antibacterial, angiogenesis	[172]
Calcium alginate	Eudragit nanoparticles containing edaravone	ROS levels (-)	NA	NA	STZ-induced diabetic rat model	One,0.5cm	Antioxidant	[173]
PVA/alginate	Green tea polyphenol nanospheres	NA	PI3K/AKT signaling pathway (+)	HFF-1 cells and HUVEC cells	STZ-induced Diabetic rat model	A 15mm thick wound on the back	Promote healing	[174]
PVA/alginate	reduced graphene oxide, Tb^3+^	Re-epithelialization and collagen deposition (+); inflammation (-)	NA	NA	STZ-induced diabetic rat model	Four, 0.8 cm	high mechanical strength, resist to protein absorption	[175]
Alginate/bioglass	SA microparticles containing conditioned medium of cells	Vascularized granulation tissue (+); inflammatory response, fibrosis, scar (-)	NA	L929 cells, Mouse aortic endothelial cells, RAW 264.7 cells	STZ-induced diabetic rat model	One, 1cm	Injectable; drug delivery by wound stage	[176]
Calcium alginate	Rubidium	Angiogenesis, re-epithelialization and collagen deposition (+)	VEGF, the nuclear factor (erythroid-derived 2)-like 2 (NRF2)/ heme-oxygenase-1(HO-1) signaling pathway (+)	HUVECs and human skin immortalized keratinocytes, fibroblasts	STZ-induced diabetic rat model	Four ,1.2 cm	Promote healing	[177]
Sodium alginate	Zn^2+^, histidine	Cell migration and angiogenesis (+)	Col 1α and VEGF (+); TNF-α and IL6(-)	Embryonic fibroblast (NIH3T3)	The db/db mice	Two ,1cm	Good injectability, biocompatibility, adhesivity and antibacterial activity	[178]
Calcium alginate	Primary macrophages and their secretome	NA	NA	L-929 cell, the RAW 264.7 cell line	The db/db mice	Two ,0. 6cm	Promote healing	[179]
chondroitin sulphate, sodium alginate, poloxamer-407	Curcumin	Tissue regeneration, collagen fibers deposition, angiogenesis, hair follicles, sebaceous glands (+); inflammation (-)	NA	3T3-L1 fibroblast cells	STZ-induced diabetic rat model	One,2 cm	Promote healing	[180]
Sodium alginate, Pectin	Simvastatin	Re-epithelialization, collagen deposition, angiogenesis (+)	NA	NA	STZ-induced diabetic rat model	Two ,0.8cm	Promote healing	[181]
Alginate	PRP	NA	NA	HaCaT keratinocytes, HDF, L929 murine fibroblast cell	The db/db mice	One,0.8 cm	Injectable, bioactive, degradable, promote healing	[182]
Calcium alginate	Deferoxamine and copper nanoparticles	NA	HIF-1α and VEGF (+)	HUVECs	STZ-induced diabetic rat model	One,1 cm	Pro-angiogenesis, antibacterial, biocompatible, antibacterial properties	[183]
oxidized sodium alginate, dopamine	NA	Angiogenesis, collagen deposition (+); inflammation response (-)	NA	L929 cells, HUVECs	STZ-induced diabetic rat model	One, 1.8 cm	Biocompatibility, suitable adhesion, promote healing	[184]

“(+)” represents upregulated or promoted

“(-)” represents downregulated or suppressed

**Fig. 8:**
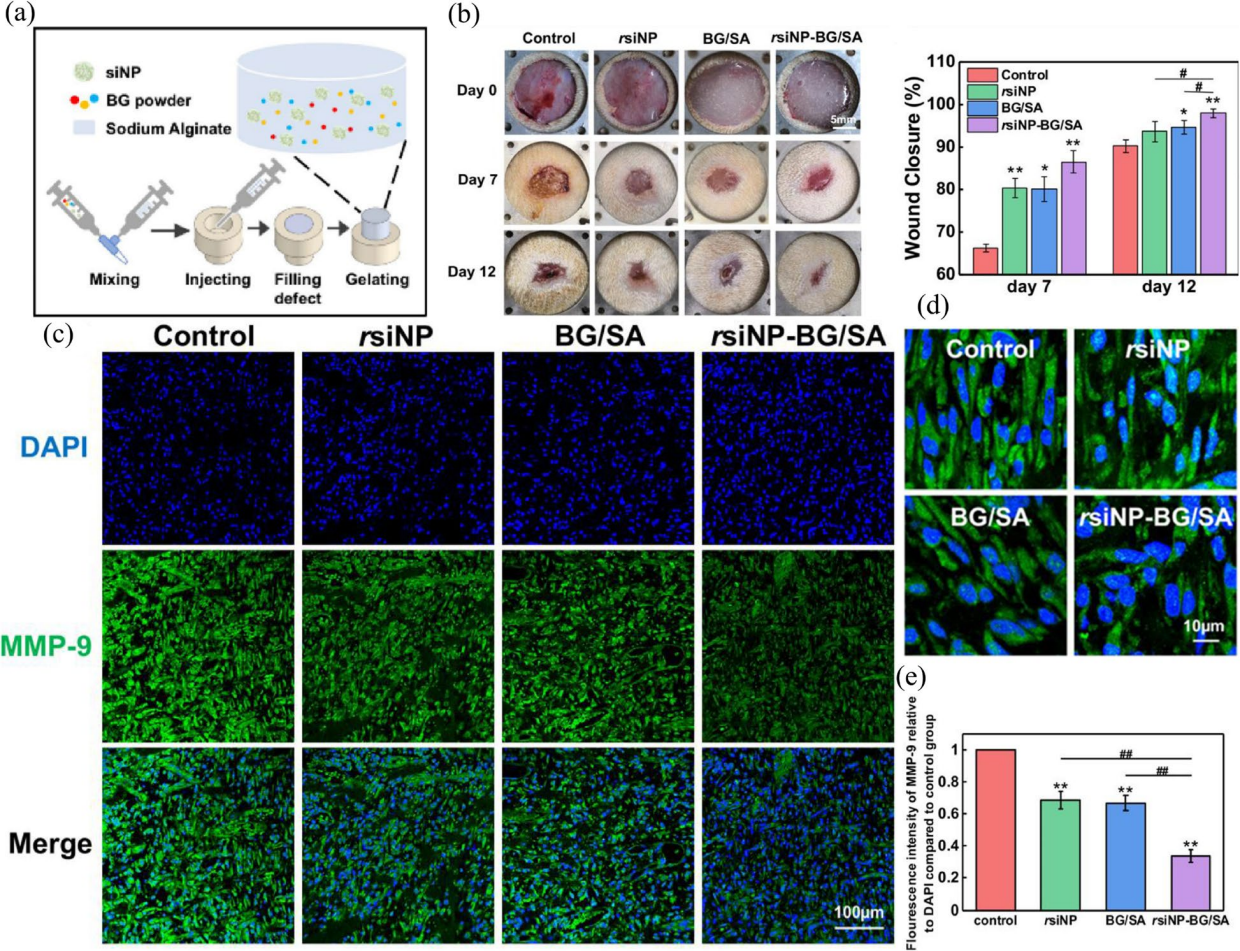
Injectable bioglass/sodium alginate (BG/SA) hydrogel loaded with MMP9-siNPs. (a) Schematic diagram of siNP-BG/SA hydrogel preparation. (b) Gross observation images and wound closure rates of wounds in the control, rsiNP, BG/SA, and rsiNP-BG/SA groups on days 0, 7, and 12. (c) Low magnification observation of MMP-9 IF staining of wound tissues. (d) High magnification observation of MMP-9 IF staining of wound tissues. (e) Quantitative statistics of MMP-9-positive areas in wound tissues. Reproduced from [185] with permission from Elsevier Copyright 2022

The most useful property of alginates is their ability to react with cations (especially calcium ions and sodium ions) to produce strong gels or insoluble polymers. Wang et al. developed a pH-responsive calcium alginate hydrogel loaded with protamine nanoparticles and hyaluronan oligosaccharide (HAO). Ca-alginate is used as a pH-sensitive dressing because of its increased abilities to swell and disrupt hydration in alkaline wound fluid in DUs. Mediated by protamine nanoparticles, this hydrogel showed significant inhibition of both gram-positive and gram-negative bacteria, reducing the chronic inflammation caused by the bacteria at the wound site. In addition, the hydrogels promoted angiogenesis in diabetic wounds through HAO-mediated enhancement of vascular endothelial growth factor (VEGF) expression, thereby accelerating the wound healing process [172]. Excessive inflammation and high expression of MMP-9 are thought to be among the characteristics of chronic diabetic trauma. Therefore, Li et al. developed an injectable 45S5 Bioglass® (BG)/sodium alginate (BG/SA) hydrogel that is loaded with small interfering RNA of MMP9 (MMP9-siRNA) (Fig. 8 (a)). Through the combined action of MMP9-siRNA and BG ion products, the hydrogel inhibited inflammation, reduced MMP9 expression, promoted collagen deposition and angiogenesis, and significantly accelerated healing of total excisional wounds in diabetic rats (Fig. 8 (b-e)). Unlike previous studies in which bioactive substances were added directly to the hydrogel, this study used a "gene silencing" approach to inhibit the expression of MMP-9 and indirectly promote skin regeneration. More importantly, by simply changing the type of siRNA, this hydrogel system can be used to enhance the regeneration of a variety of damaged tissues with an imbalance in ECM synthesis and degradation, thus demonstrating its wide application potential [185].

In recent years, semisynthetic hydrogels combining alginate and synthetic polymers have been favored. Chen et al. synthesized novel tea polyphenol nanospheres (TPNs) and encapsulated them in PVA/alginate hydrogels (TPNs@H). Animal experiments and molecular mechanistic studies demonstrated that TPNs@H could promote wound healing in diabetic rats by regulating the PI3K/AKT signaling pathway [174]. Wang et al. prepared PVA-alginate hydrogels with high mechanical strength and resistance to protein uptake and loaded them with reduced graphene oxide (rGO) and ions of the rare earth metal terbium (Tb^3+^). Antibacterial experiments showed that the PVA-SA-rGO-Tb^3+^ hydrogels inhibited S. aureus and P. aeruginosa biofilms, and the antibacterial activity improved by 3.5 and 2 orders of magnitude compared with the PVA-SA-rGO and PVA-SA-Tb^3+^ hydrogels, respectively. More importantly, since the antibacterial effects of the PVA-SA-rGO-Tb^3+^ hydrogel are independent of antibiotics, there is no need to worry about the development of organismal drug resistance [175].

It is important to note that the hydrogels in the above study (both natural and synthetic) were synthesized based on long-chain polymers. As a result, these hydrogels cannot be used directly for tissue regeneration, and they take at least a few days to degrade in the wound, which may prolong the regeneration time. Yao et al. therefore developed a new healing hydrogel based on histidine. Histidine is cross-linked with zinc ions (Zn^2+^) and sodium alginate (SA) via dynamic coordinate and hydrogen bonds. Histidine is cross-linked with zinc ions (Zn^2+^) and sodium alginate (SA) via dynamic coordinate and hydrogen bonds, respectively, forming a histidine-sodium alginate-Zn^2+^ (denoted as HSZH) hydrogel. In vivo experiments showed that the mean healing time in the HSZH group was approximately 13 days, while the complete closure time in the control group was approximately 27 days. The HSZH hydrogel with double dynamic key crosslinking has a strong ability to promote wound healing, and the healing rate is more than 100% higher than that of the control group. This work provides a new strategy for designing wound dressing materials in which weakly cross-linked materials based on tissue-friendly small molecules heal wounds more effectively than highly cross-linked materials based on long-chain polymers [178].

### Gelatin-based hydrogels

Gelatin is a product of the partial denaturation and hydrolysis of collagen, and is widely used in biomedical fields because of its good biodegradability and histocompatibility. Unlike collagen, gelatin is generated by the thermal denaturation of collagen, so it has lower immunogenicity than collagen and is less likely to cause an immune response in the body [186]. The arginine-glycine-aspartate (RGD) cell binding sequence and matrix metalloproteinase (MMP) reactive peptide sequence are retained in the gelatin fabrication process. Due to the presence of these two sequences, the gelatin matrix promotes cell adhesion mediated by integrins as well as enzymatic degradation [187].

In 2000, Van den Bulcke et al. first reported modifying the amino side chains of gelatin with methacrylic anhydride to produce methacrylamide-based gelatin (GelMA) [188]. Due to the presence of methacryloyl, GelMA has photocrosslinking properties. When a photoinitiator is added, the aqueous solution of GelMA immediately crosslinks under UV light to produce a GelMA hydrogel with excellent thermal stability. The most useful attribute of GelMA is its highly tunable mechanical properties. GelMA hydrogels with different mechanical strengths can be obtained by varying the crosslinking conditions, including the GelMA polymer concentration, degree of methacrylation, wavelength and intensity of the light applied, and light exposure time [189].

**Fig. 9:**
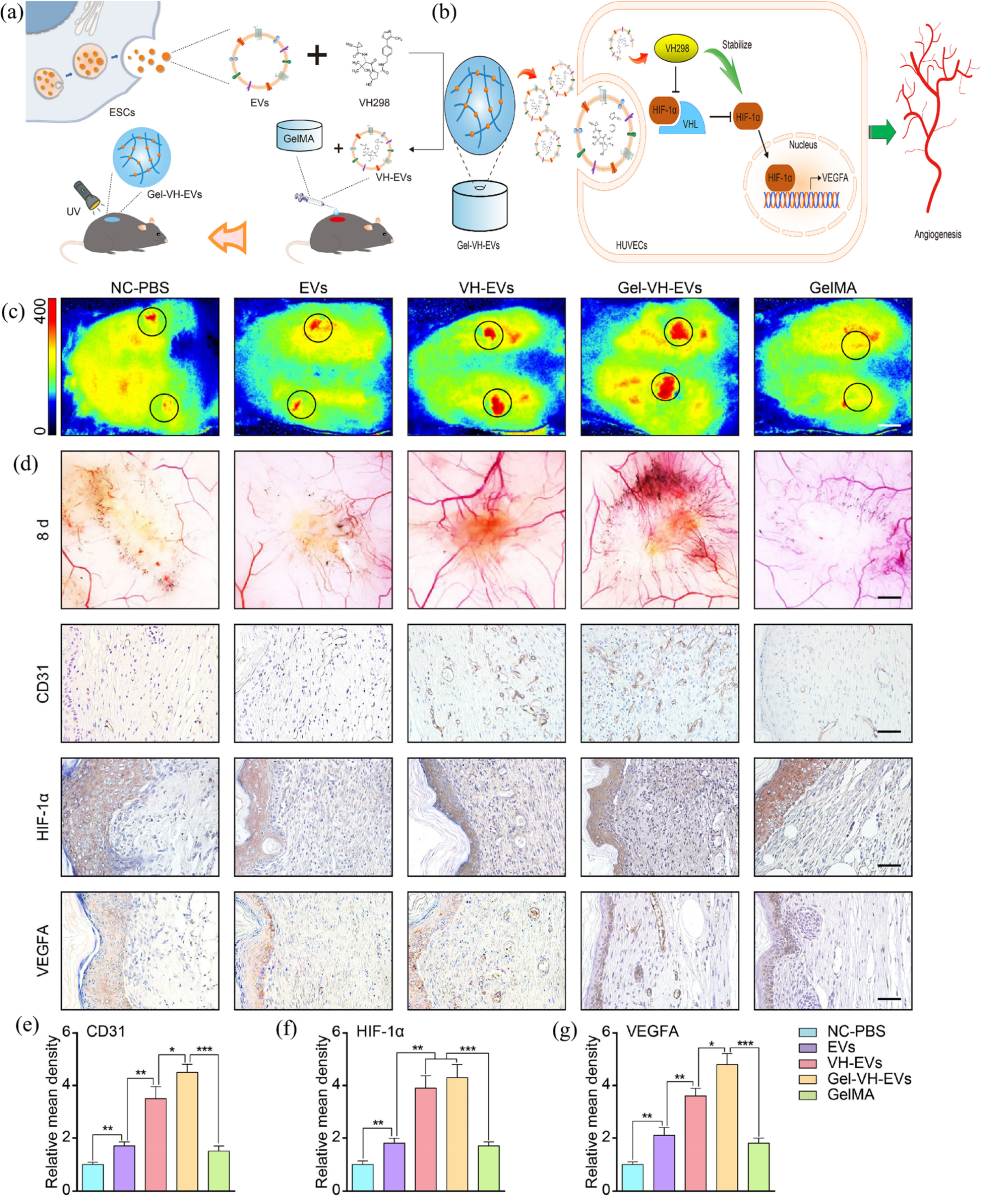
A GelMA hydrogel loaded with VH-EVs for the treatment of diabetic wounds. **a** Schematic illustration of the experimental procedure of the in vivo study. **b** Underlying mechanisms of VH-EVs released from GelMA hydrogel for enhancing angiogenesis by stabilizing HIF-1α in HUVECs. **c** Blood perfusion at the wound areas of different groups was evaluated by a laser Doppler perfusion imaging system. **d** The gross view of neovascularization toward the wound in different treatment groups. **e** Immunohistochemical staining of CD31, HIF-1α, and VEGFA in regenerated skin tissue in different groups at day 12. **e-g** Quantitative analysis of the relative mean density of immunohistochemical staining of CD31, HIF-1α, and VEGFA in regenerated skin tissue in different groups at day 12. Reproduced from [190] with permission from Elsevier Copyright 2022

Since chemical modification of gelatin with methacrylic anhydride usually involves only amino acid residues with a molar ratio of less than 5%, the RGD sequence and MMP sequence are retained. Thus, GelMA still has good cell adhesion and proliferation properties and enzymatic degradation abilities and can be widely used in the treatment of DUs **(**Table 4**)**. Wang et al. prepared a GelMA hydrogel loaded with VH-EVs for the treatment of diabetic wounds. VH-EVs are formed by extracellular vesicles from epidermal stem cells coated with VH-298 (a stabilizer of HIF-1α) (Fig. 9 (a)). Because VH-EVs are cleared quickly from the body, they need to be injected around the wound frequently or intravenously, resulting in waste of VH-EVs and the risk of bleeding and infection. GelMA provides VH-EVs with continuous release, superior bioavailability and a faster degradation rate. In diabetic mice, Gel-VH-EV hydrogel effectively promoted wound healing by locally enhancing blood supply and angiogenesis (Fig. 9 (b)). The mechanism of promoting angiogenesis may be related to the activation of the HIF-1α/VEGFA signaling pathway (Fig. 9 (c-g)). Notably, although many studies of exosomes from other stem cells have been reported previously, few studies have focused on the effects of ESC-derived EVs on skin regeneration. The ESC-EVs used in this study were shown for the first time to contribute to the proliferation, migration and tubular formation of HUVECs and to the treatment of diabetic wounds, which carries out a new stem cell strategy for the treatment of diabetic wounds [190]. Alap Ali Zahid et al. developed a composite hydrogel that provides a continuous supply of nitric oxide (NO). They incorporated the NO donor S-nitroso-N-acetyl penicillamine (SNAP) into a highly porous GelMA hydrogel to improve wound healing in diabetic wounds. In the diabetes rat model, compared with the blank GelMA hydrogel and the control group, the GelMA patch with a SNAP concentration of 0.08% (w/w) has obvious wound contraction and faster wound healing [191].

**Table 4:** Gelatin based hydrogel

System	Substance	Mechanism	Signal pathway	Cell line	Animal	wound size	characteristic	Ref.
GelMA	CONPs	Angiogenesis (+); free radical (-)	NA	HaCaT keratinocytes and 3T3 fibroblasts	STZ-induced diabetic rat model	Four ,1 cm	Promote healing	[192]
GelMA	SNAP(a nitric oxide donor)	Cell proliferation and migration, angiogenesis (+); the growth of bacteria (-)	NA	3T3 fibroblasts and HaCaT keratinocyte cells	STZ-induced diabetic rat model	Four ,1 cm	Promote healing	[191]
Gelatin methacrylamide	4-octyl itaconate -modified black phosphorus nanosheets	Antioxidant activity, neovascularization (+)	SOD2, VEGF, FGF-2, CD31, α-SMA (+)	HUVECs	STZ-induced diabetic rat model	One, 1.5 cm	Antibacterial, antioxidant, combine with PDT and PTT, Near-Infrared Responsive Properties	[193]
Aminated gelatin, oxidized dextran	Nano-ZnO, Pf-encapsulated micelles	Angiogenesis and collagen deposition (+); inflammation (-)	IL-10, CD31(+); TNF-α (-)	Murine L929 fibroblasts, HUVECs	STZ-induced diabetic rat model	Four, 1.2 cm	Sequential hemostatic, microbe killing, angiogenic, injectable, inflammation-responsive, self-healing	[194]
Gelatin, 3-carboxy-phenylboronic acid, PVA	VAN-AgNCs and micelles loaded with nimesulide (NIM)	New vessel formation, collagen deposition (+); inflammation (-)	IL-10(+); TNF-α (-)	Mouse fibroblast L929, HUVECs	STZ-induced diabetic rat model	Four ,1.2 cm	Antibacterial and anti-inflammatory, biocompatibility, hemostasis, inflammation-responsive	[195]
methacryloyl-substituted Bletilla Striata polysaccharide, gelatin	NA	The proliferation and migration of fibroblasts, angiogenesis (+)	The transition of macrophages from M1 to M2(+)	NIH/3T3 cells, RAW 264.7 cell	STZ-induced diabetic rat model	NA, 0.6 cm	Promote healing, porous, biodegradable, adjustable mechanical properties	[196]
GelMA, oxidized chondroitin sulfate	OCS-polypyrrole conductive nanoparticles	Neurovascular regeneration, collagen deposition (+)	PI3K/AKT and MEK/ERK pathways (+)	Fibroblasts, HUVECs	The SD mice	Four,1.2 cm	Promote healing	[197]
GelMA	Cerium containing bioactive glass	Granulation tissue, collagen deposition, angiogenesis (+)	CD31, α-SMA (+)	L929 cells, HUVECs	STZ-induced diabetic rat model	One, 1 cm	Excellent antibacterial, angiogenic, cell compatibility	[198]
Polyacrylamide, gelatin, and ε-polylysine	NA	Collagen deposition, angiogenesis (+); bacterial breed (-)	CD31(+)	L929 cells	STZ-induced diabetic rat model	One, 1 cm	super-stretchability, enduring water retention, adhesiveness, persistent antibacterial property	[199]
GelMA	VH298-loaded extracellular vesicles	Blood supply, angiogenesis (+)	HIF-1α mediated enhancement of angiogenesis (+)	ESCs and HUVECs	The db/db mice	Two,1 cm	High biocompatibility, proper mechanical properties, promoting angiogenesis	[190]
GelMA	Adenine acrylate, and CuCl_2_	Epithelialization, collagen deposition angiogenesis (+) proinflammatory factors (-)	α- SMA and CD31 (+); IL-6(-)	Fibroblast cell lines (L929 cells)	STZ-induced diabetic rat model	NA, NA	Good biocompatibility, antibacterial properties, promote healing	[200]

“(+)” represents upregulated or promoted

“(-)” represents downregulated or suppressed

Combining gelatin with other polymers has been explored for a variety of purposes, including enhancing mechanical properties, modifying a structure or imparting specific attributes. Fan et al. prepared a conductive hydrogel composed of gelatin methacryloyl, oxidized chondroitin sulfate (OCS), and OCS-polypyrrole conductive nanoparticles. Conductive hydrogels combine the advantages of hydrogels and conducting polymers (CPs) to provide suitable electrical conductivity and mechanical properties for neurovascular regeneration. In vitro and in vivo studies have shown that the hydrogel can induce neurovascular regeneration by increasing the intracellular Ca2+ concentration, thereby promoting protein phosphorylation in the MEK/ERK and PI3K/AKT pathways. The wound model of diabetic rats further demonstrated that conductive hydrogels based on ECM can promote collagen deposition and diabetic wound regeneration. Compared with previous studies, this study provides us with a new idea to promote diabetic wound neurovascular regeneration [197]. Guo et al. prepared a sequential hemostatic, antibacterial, and proangiogenic injectable hydrogel based on the complex microenvironment of diabetic wounds. The hydrogel was made by mixing ethylenediamine-modified gelatin (N-Gel) with oxidized dextran (ODex) containing abundant aldehyde groups. Gelatin itself has hemostatic properties, and the formation of a dynamic Schiff base gives the hydrogel pH-responsive properties. Piggybacked zinc oxide nanoparticles (nZnO) and Pf-encapsulated micelles endowed the hydrogel with antibacterial and proangiogenic abilities, respectively. After release of the antimicrobial drug nZnO, the Pf-encapsulated micelles responded to the acidic and ROS-rich environment of chronic infections, achieving on-demand drug release. These continuous activities allowed the hydrogel to adapt to phase changes during wound healing and achieve better healing effects [194].

GelMA as a tissue engineering biomaterial has attracted more and more attention in recent years, because GelMA provides biological support for cells and maintains specific physical and chemical properties. There are many laboratory studies on GelMA hydrogels, but there are still many problems before clinical application, including the standard of GelMA synthesis, the standard of gel time, and the safety standard of hydrogel materials. In addition, the use of photoinitiator is unavoidable in order to synthesis hydrogel. We need to ensure that all introduced ingredients are non-toxic and that the degradation components of the hydrogel dressing do not negatively affect the system. We believe that with the continuous development of research, GelMA hydrogel and its composites will surely play a greater role in repair, tissue engineering, controlled release of drugs and other fields.

### Cellulose-based hydrogels

Cellulose is one of the most widely distributed and abundant polysaccharides in nature. In 1838, the French chemist Anselme Payen isolated cellulose from plant fibers using nitric acid and determined its chemical structure [201]. Cellulose is a linear polymer composed of D-glucose units connected by β-1,4-glycosidic bonds [202]. There are three free hydroxyl groups on each glucose unit, located on three carbon atoms, C2, C3 and C6, and these active alcoholic hydroxyl groups are a double-edged sword for the application of cellulose. On the one hand, the simple and homogeneous repeating unit in cellulose contains a large number of alcoholic hydroxyl groups, which leads to a large number of hydrogen bonds that can form between cellulose molecules. This makes cellulose neither water soluble nor easily soluble in organic solvents, which largely limits the development and application of cellulose-based functional materials. On the other hand, the free alcoholic hydroxyl groups can be used to modify the cellulose, which can improve its processing performance and allow cellulose materials with different applications and functions to be obtained.

Modified cellulose is a hydrophilic material synthesized by the separation and ring opening of polysaccharide hydroxyl groups and propylene oxide under strong alkaline conditions. Commonly used modified cellulose materials include carboxymethyl cellulose (CMC), hydroxypropyl cellulose,and hydroxypropyl methyl cellulose, among which CMC is widely used in wound dressings due to its good biodegradability, histocompatibility, and ability to promote fibroblast proliferation and migration. Regranex® (Becaplermin Gel), a mixture of recombinant human platelet-derived growth factor (rh-PDGF) and carboxymethyl cellulose (CMC), is currently the only FDA-approved hydrogel for the treatment of DFU. Regranex® promotes DU wound healing by promoting fibroblast proliferation, collagen deposition, and accelerated neovascularization. A multicenter, double-blind randomized controlled trial showed that, compared to a placebo gel, Regranex® 100 ug/g significantly increased the incidence of complete wound closure by 43% and decreased the time to achieve complete wound closure by 32% [203]. Despite promising clinical trials, Regranex^®^ gel has shown limitations in clinical practice, with a 15-gram tube of hydrogel costing about $1,300, making it unaffordable for many diabetic ulcers.

**Fig. 10:**
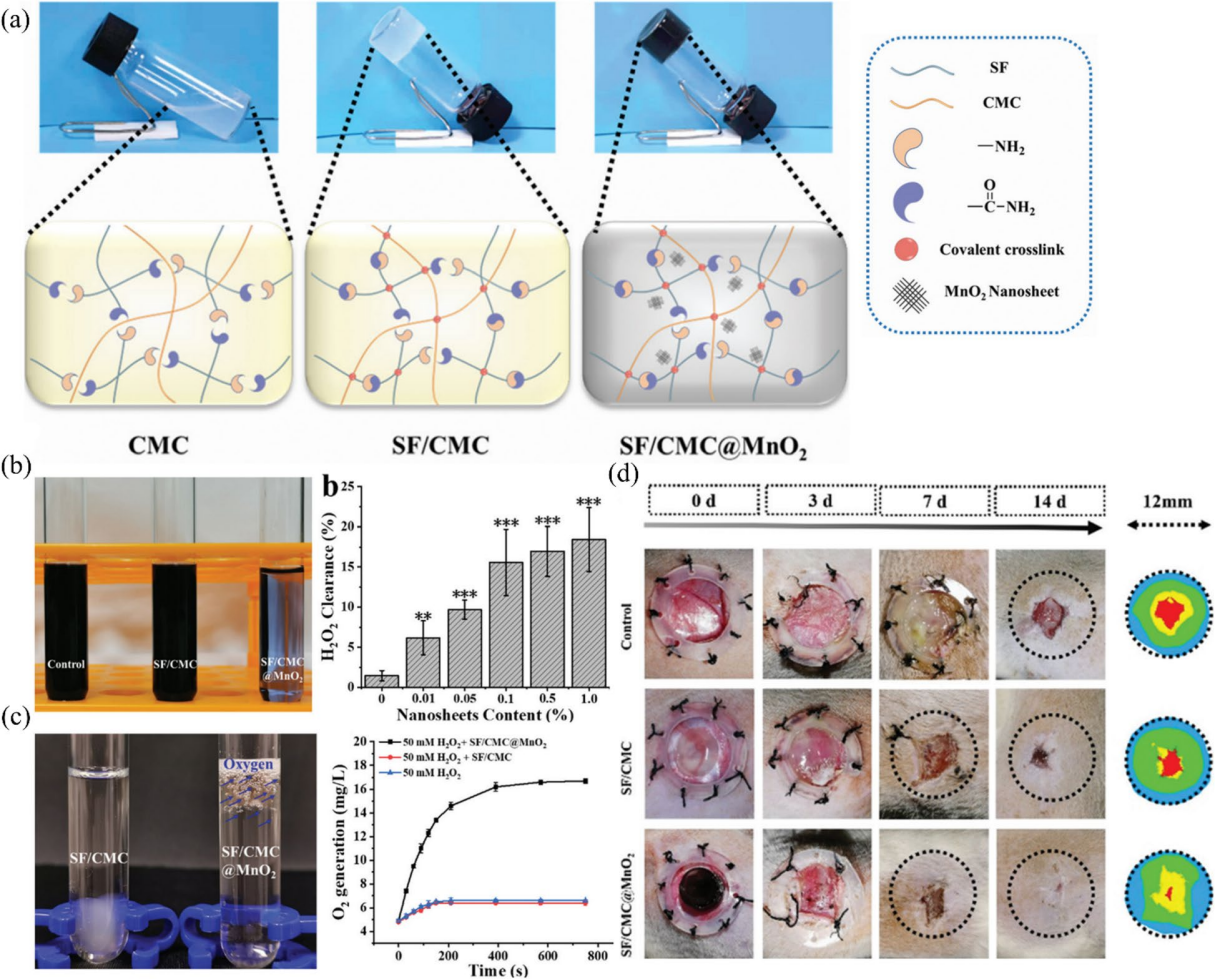
Continuous oxygen-supplying hydrogel based on hydroxymethyl cellulose and silk fibroin, MnO2 nanosheets. **a** The preparation process of hydrogels. **b** The H2O2 clearance rate increased with increasing Mn2+ nanosheet content. **c** Effects of different types of hydrogels and O2 generation with H2O2 solution. **d** Images of the wounds in diabetic mice after treatment with commercial dressing films (3 M, Tegadermt), SF/CMC hydrogels and SF/CMC@MnO2 hydrogels for 0, 3, 7 and 14 days. Reproduced from [205] with permission from RSC Publishing Copyright 2022

To better help patients, scientists are conducting more research on hydrogels to treat DU. Wang et al. prepared a new composite hydrogel consisting of carbomer 940 (CBM) and CMC loaded with natural polysaccharides derived from the herbal residue of Periplaneta americana. The ethanol extract of P. americana has been developed into a clinical patent liquid preparation, Kangfuxin, which was approved by the China Food and Drug Administration and has been used to treat various skin or mucosa injuries for over 40 years. In vivo experiments showed that the hydrogels accelerated wound healing in a diabetic model rat by promoting wound closure, collagen deposition, M2 macrophage polarization and angiogenesis [204]. Pu et al. developed a continuous oxygen-supplying hydrogel based on hydroxymethyl cellulose and silk fibroin, MnO2 nanosheets (Fig. 10 (a)). Because of the presence of hydroxymethyl cellulose, the problem of the secondary structure transformation of SF generated large b-sheet domains, which significantly enhanced the brittleness of the hydrogels. At the same time, the inherent shear thinning effect gives the dual-network hydrogel satisfactory injection performance and can be tailored to a predetermined shape to accommodate irregularities in diabetic wounds. In contrast to most strategies that rely on biomolecular delivery to promote wound healing, the researchers used the synergistic effect of the uniformly dispersed ROS scavenger MnO2 and MMP-sensitive SF to convert excess ROS in situ to the required O2 to shorten inflammation while downregulating the expression of MMPs to promote matrix remodeling, which plays a role in different stages of wound healing (Fig. 10 (b-c)). Significantly, the healing rates of SF/CMC@MnO2 hydrogels at 7 and 14 days were 76% and 100%, respectively, significantly faster than the healing rates at 7 and 14 days for commercially available dressings (3 M, TegadermtTM) (30% and 80%, respectively), demonstrating great promise for the clinical application of SF-based hydrogels (Fig. 10 (d)) [205].

In recent years, the preparation of high-performance composite hydrogels by using highly crystalline, hard cellulose for reinforcement has become common. Wang et al. successfully constructed highly efficient self-healing hydrogels by forming dynamic covalent Schiff base bonds between aldehyde-modified methyl-cellulose (MC-CHO) and chitosan-grafted poly(ethylene glycol) (CS-g-PEG). Due to the presence of Schiff base bonds, the initial shape of the gel network is conserved at low strain, while the gel network is broken at large strain. When the strain oscillation returned to a lower value (1%), both modules immediately returned to their initial values, proving that the hydrogel network recovered rapidly after failure. After three cycles, the recovery G′ of the hydrogels was still equivalent to the primary value, indicating that the hydrogel networks were repeatable with the same efficiency. The addition of placental mesenchymal stem cell-derived exosome (PMSC-Exo) endosomal hydrogels with the properties of promoting angiogenesis and inhibiting apoptosis constructed a new platform for diabetic wound healing [206].

Notably, some properties of cellulose hydrogels can indeed relieve the limitations of existing hydrogels to some extent **(**Table 5**)**, but there are still some problems to be solved. First, the network structure of traditional cellulose hydrogels has no stress dispersion mechanism, and small external forces can cause the destruction and collapse of the hydrogel structure. Second, although researchers have been able to improve the mechanical strength of cellulose hydrogels by crosslinking or introducing reinforcing components, these two strategies are cumbersome and will affect the biocompatibility and degradation of hydrogels to varying degrees [201]. Therefore, researchers need to further prepare and develop tough and strong cellulose gels and balance the relationship between the characteristics of various hydrogels.

**Table 5:** Cellulose-based hydrogel

System	Substance	Mechanism	Signal pathway	Cell line	Animal	Wound size	characteristic	Ref.
Carbomer 940, CMC	Periplaneta americana herbal residue	Wound closure, collagen deposition, angiogenesis (+)	TNF-α, IL-10, VEGF, the transition of macrophages from M1 to M2(+)	3T3 fibroblast	STZ-induced diabetic rat model	One, 2 cm	Porous structure, good rheological property, swelling capability, biocompatibility	[206]
Oxidized CMC	Polyethyleneimine and tobramycin	Granulation tissue, collagen deposition, angiogenesis (+)	Arg-1(+); F4/80, iNOS, TNF-α(-) ,	NCTC clone 929 cells (L cells, L-929), HUVECs	STZ-induced diabetic rat model	One, 1 cm	Anti-inflammation, anti-infection, injectable, self-healing, biocompatible properties, pH-sensitive	[207]
silk fibroin, CMC	MnO2 nanosheets	ECM integration, angiogenesis(+); inflammatory level (-)	NA	L929 cells	STZ-induced diabetic rat model	One, 1 cm	Injectable, biocompatible, ROS scavenging, sustained O2 generation	[205]
Hydroxypropyl methylcellulose	Val loaded solid lipid nanoparticles	NA	COX-2, NF-κB, NO, TGF-β, VEGF, MMPs (+)	NA	STZ-induced diabetic rat model	A layer of the skin in 3x5 mm thickness wound on the foot’s dorsal surface	Anti-infection	[208]

“(+)” represents upregulated or promoted

“(-)” represents downregulated or suppressed

### Composite hydrogels

Polysaccharides such as chitosan, sodium alginate and HA are soft in nature, and if used alone to prepare hydrogels, the gel materials are soft and weak and display poor mechanical properties and low mechanical strength [23]. If synthetic materials such as PVA and PEG are used in combination to prepare hydrogels, although the mechanical strength is closer to ideal, the biocompatibility is poor and the hydrogels do not easily degrade [4]. Therefore, some researchers have combined two or three different natural polysaccharides to produce hydrogels with the best mechanical strength and optimal degradation rate by adjusting the ratio and type of modification. Table 6 shows the major composite hydrogels used for DUs over the last five years.

**Table 6:** Composite hydrogel

	System	Substance	Mechanism	Signal pathway	Cell line	Animal	Wound size	Characteristic	Ref.
HA and CS	OHA and CMC	Curcumin, EGF	Collagen deposition, angiogenesis (+); oxidative stress, inflammation (-)	IL-6, TNF-α, IL-1β, MMP9 (-)	NIH-3T3 cells	STZ-induced diabetic rat model	One,0. 8cm	On-demand drug release, superior rheological, swelling, degradation, biocompatibility, antibacterial, hemostatic properties.	[209]
	Quaternary ammonium chitosan and OHA	α-LA	Cell proliferation, neovascularization(+)	PCNA, Ki67, CD34, α-SMA (+)	Human dermal fibroblasts, HaCAT, HUVECs	STZ-induced diabetic rat model	Three ,1.5cm	Superior injectability, self-healing, adhesive mechanical properties, antibacterial, biocompatibility	[210]
	OHA and SCS	Insulin-loaded micelles and EGF	Fibroblast proliferation, collagen deposition, myofibrils (+)	NA	3T3 cells	STZ-induced diabetic rat model	One,0. 8cm	pH-responsive, low biological cytotoxicity, good biocompatibility	[211]
	N-carboxyethyl chitosan, HA-ALD	BM-MSCs	Granulation tissue formation, collagen deposition, nucleated cell proliferation, neovascularization (+)	TGF-β, VEGF, bFGF, CD31, Ki67(+)	BM-MSCs	STZ-induced diabetic rat model	one,0. 5cm	Promote stem cell proliferation or secretion of growth factors, injectable, self-healing properties	[212]
	CMCS and OHA	Taurine	Anti-inflammatory activity, angiogenesis (+)	VEGF (+); TNF-α, IL-6(-)	L929 cells	STZ-induced diabetic rat model	One,0. 8cm	Self-healing, pH- responsive, good biocompatibility, mechanical properties	[213]
	OHA and CMCS	Au-Pt alloy nanoparticles	Angiogenesis, epidermis growth, collagen deposition (+); Inflammation (-)	VEGF, CD31, IL-10 (+); IL-1β (-)	NA	STZ-induced diabetic rat model	Three, 1.2 cm	Self- healing, regulating wound pathological microenvironment, antibacterial	[214]
Cellulose and chitosan	CMCS-Hep and CMC-A	SOD, rhEGF	Cell migration and proliferation, collagen fibers deposition, blood vessels (+); DNA damage, inflammation (-)	ki67, CD31(+)	L929, HUVEC	STZ-induced diabetic rat model	One,1 cm	biodegradable, injectable, self-healing, and low-toxic	[215]
	Methylcellulose, chitosan	Exosomes from PMSC cell culture	Angiogenesis (+); apoptosis (-)	Bcl-2, VEGF (+); Bax(-)	293T cells	C57BLKS-Leprdb mice diabetes model	NA,0.7cm	Self-healing, injectable, biocompatibility	[206]
Gelatin and chitosan	Gelatin and chitosan	Histatin-1, polypyrrole based conductive nanoparticles	the deposition of ECM, vascularization (+), inflammatory response, cell damage (-)	CD31, α-SMA (+); TNF-α (-)	L929 cells, HUVECs	db/db congenital diabetic mice (type II)	One,1cm	Good adhesion, stability, and biocompatibility, conductivity	[216]
Chitosan and alginate	CMCS and SA	Ir-fliq probe	Bacterial growth (-)	NA	NIH3T3, RAW264.7 and H22 cells	STZ-induced diabetic rat model	One,0.5cm	Real-time monitoring	[217]
Cellulose and HA	OHMPC, HA-ADH	Alg@ori, HA-PEI@siRNA-29a	Angiogenesis (+), inflammation (-)	α-SMA and CD31(+); IL-6 and TNF-α (-)	Mouse fibroblasts L929 cells	STZ-induced diabetic rat model	Wounds on the dorsal region	Anti-inflammation, angiogenesis, excellent biocompatibility	[218]

“(+)” represents upregulated or promoted

“(-)” represents downregulated or suppressed

## Bioactivators for DUs

Wound hyperglycemia-mediated vasculopathy and neuropathy can cause decreased vasoconstriction and diastolic function and reduced self-regulation of skin blood flow, causing impaired blood circulation in the distal limb. In addition, persistent chronic inflammatory reactions, as well as microbial infections, cause impaired neovascularization, thus limiting the transport of oxygen and nutrients. Therefore, certain substances need to be delivered to accelerate the healing of diabetic wounds. In this section, we will provide a further overview of the various active ingredients used in the treatment of DFUs and their functions and mechanisms of action** (**Table 7**).**

**Table 7:** Bioactivators for diabetes ulcer

	Substance	Treatment of type	Outcome	Ref.
Cells/Exos	AD-MSCs	MSC transplantation	Antibacterial, hasten healing	[219]
	Exosomes from ADSCs	Exosomes transplantation	Accelerate angiogenesis	[220]
			Promote angiogenesis via miR-106a-5p and FGF4/p38MAPK pathway	[221]
	Extracellular vesicles from HF-MSCs	Exosomes transplantation	Increase HDFs proliferation, angiogenesis	[222]
	BM-MSCs	MSC transplantation	An increase in the length of epithelial edges, collagen content, microvessel density and a higher expression of VEGF	[223]
	Exosomes from ADSCs	Cryogels	Enhanced collagen deposition, faster re-epithelialization, increased neovascularization, and decreased oxidative stress	[224]
	ADSCs	MSC transplantation	Enhance VEGFR3-mediated lymphangiogenesis via METTL3-mediated VEGF-C m6A modification	[225]
		Cryogels	Reduce inflammation and promote collagen deposition	[226]
	ADSCs and PRP	Injection	Upregulate Notch 1 signaling, enhancing angiogenesis, and triggering epidermal cell proliferation and recruitment.	[227]
	ESCs-Exo	Subcutaneous injections	Decreasing inflammation, augmenting wound cell proliferation, stimulating angiogenesis, and inducing M2 macrophage polarization.	[228]
GFs	EPCs and aFGF	Cryogel	Enhanced granulation formation, collagen deposition, reepithelization.	[229]
	Umbilical cord SCF	Hydrogel	Promote angiogenesis and collagen deposition.	[230]
	EGF	Nanoparticles	Thorough re-epithelization, reduced inflammatory response, faster collagen deposition, and advanced collagen maturation	[133]
		Patch	Promote the migration and proliferation of multiple types of cells (keratinocytes, fibroblasts, and endothelial cells) and enhance angiogenesis	[231]
		Coacervates	Recovered horizontal migration of epidermis over the newly deposited dermal matrix, reduced levels of proinflammatory cytokines IL-1, IL-6, and THF-α	[232]
	VEGF and PDGF-BB	Hydrogels	Induce angiogenesis and arteriogenesis	[233]
	VEGF	Hydrogel patch	Downregulating the expression of inflammatory factors, promoting collagen deposition and angiogenesis	[234]
Medicines	Metformin	Microneedles	Inhibiting inflammation, providing oxygen, absorbing excess exudate	[235]
	Ferulic Acid	Nanofibers	Antibacterial, epithelial layer regeneration and collagen formation	[236]
	Cefotaxime sodium	Hydrogel membranes	Angiogenesis, accelerated reepithelization, collagen deposition	[237]
			Angiogenesis, mature collagen deposition and epidermis regeneration, antibacterial	[148]
	DCH, CEX	Nanofibers	L929 fibroblasts proliferation and growth, antibacterial	[238]
	Ceftriaxone, melittin	Hydrogel	Reduction in IL-6 and TNF-α, increase in hydroxyproline, VEFG-A, and TGF-β1	[239]
	L-carnosine and curcumin	Hydrogel	Inactivation of MMP-9, antibacterial	[240]
Metal	AgNPs	Topical preparations	Antimicrobial, anti-oxidative, anti-inflammatory, and angiogenesis	[241]
		Spray	Promote collagen deposition, low levels of inflammatory infiltrate	[242]
		Topical preparations	Antimicrobial, decreased the mRNA and protein expression of MMP-2 and MMP-9	[243]
		Hydrogel	Inhibits collagenase and MPO activity	[244]
	Ag^+^	Hydrogel	Thorough reepithelization, sufficient collagen deposition, and accelerated collagen maturation	[245]
	ZnO NPs	Topical preparations	Antibacterial	[246]
	CuNPs	Hydrogels	Stimulated the levels of HIF- 1α and VEGF	[183]
GoX	Gox, MnO2	Hydrogel	Antibacterial, remove biofilm, oxygen generation, angiogenesis	[247]
	GOX	Hydrogel	Antibacterial, promote healing	[248]
	GoX, hemin	Hydrogel	Eradication of infection, reduction of the glucose concentration	[249]
	DFO, Gox	Hydrogel	Induce reepithelialization, collagen deposition, angiogenesis	[250]

### Stem cells/exosomes

Stem cells are a class of cells derived from embryonic, fetal or adult tissues that have a strong capacity for self-renewal and self-replication under specific conditions and the potential to differentiate into a variety of cells, tissues and organs. In recent years, stem cell isolation and culture techniques have been developed, bringing new hope for the treatment of patients with DUs. The mechanism by which stem cells promote wound healing is still under investigation, but it is clear that stem cells differentiate into vascular endothelial cells, fibroblasts and smooth muscle cells, promoting the formation of new functional blood vessels, bringing oxygen and nutrients to the ulcerated wound, discharging the accumulated carbon dioxide and metabolic waste, and directly accelerating wound healing [251, 252]. Moreover, stem cells synthesize and secrete various cytokines, such as IL-10, VEGF and NGF, which are involved in immune regulation, angiogenesis and nerve regeneration, and indirectly promote wound healing [253]. However, the clinical application of traditional stem cell transplantation is very limited due to their low colonization rate at the injured site and potential tumorigenicity. An increasing number of studies have shown that stem cell transplantation promotes wound healing not only through direct cell differentiation but also indirectly through paracrine action. As the key mediators of paracrine action, exosomes are extracellular vesicles with diameters of 30-150 nm that contain a variety of bioactive substances, such as lipids, proteins and RNA, and can deliver these bioactive substances to recipient cells through membrane fusion or endocytosis to regulate the biological activity of the target cells [254]. In addition, exosomes have advantages that stem cells do not have, including low immunogenicity, high stability, easy storage, etc.

ADSC-Evs are the most commonly used extracellular vesicles in DUs. ADSC-Evs are widely available and easily accessible, and they accelerate the healing of DUs mainly by promoting revascularization. In a comparative study by Margherita Pomatto et al, ADSC-Evs displayed improved proangiogenic activity and accelerated DU wound healing compared to BMSC-Evs [255]. Li et al. applied ADSC-Evs overexpressing Nrf2 in a streptozotocin (STZ)-induced diabetic rat model, and compared to untreated controls, the levels of SMP30 and VEGF were significantly increased and the phosphorylation of VEGFR2 rose, while the levels of ROS and the inflammatory cytokines NOX1 and NOX4 were significantly decreased in rats treated with ADSC-Evs. These data indicated that ADSC-Evs could foster wound healing by promoting neovascularization and reducing inflammation. In addition, they found that the exosomes secreted by ADSCs promoted endothelial progenitor cell proliferation and angiogenesis in a high-glucose environment and that Nrf2 overexpression increased this effect [256]. Stem cells and exosomes are of great research value, but there are still some problems that should be noted: stem cells are vulnerable to ischemic and hypoxic microenvironments, and the secretion and purification of exosomes remain challenging. Therefore, most studies are currently in the preclinical stage, and more research is needed in the future to explore the production and scope of adaptation of stem cells.

### GFs

GFs are active proteins or peptides present in living organisms that have a wide range of regulatory effects on growth and development. Most GF receptors exhibit tyrosine protein kinase activity, and when GFs bind to their receptors, they can act through the RTK-Ras-MAPK pathway. VEGF, for example, is a highly specific pro-vascular endothelial GF with the highest activity and specificity among known angiogenic factors. Studies have shown that VEGF binds to VEGFR on the endothelial cell membrane, causing autophosphorylation of the receptor, which in turn leads to the conversion of the inactive Ras-GDP complex to the active Ras-GTP complex, thereby activating MAPK, inducing endothelial cell proliferation and accelerating neovascularization [257, 258].

In recent years, an increasing number of researchers have topically applied GFs (i.e., FGF, EGF, VEGF, PDGF) to DFU patients and achieved good therapeutic effects. However, the high concentration of proteolytic enzymes on the wound surface, sudden release of GFs and susceptibility to codelivered substances have greatly limited the application of GF [4]. Lee et al. combined perfluorocarbon emulsions, epidermal growth factor (EGF)-loaded chitosan nanoparticles, and polyhexamethylene biguanide (PHMB) in a chitosan hydrogel, which was given the name PEENPPCH. This hydrogel can promote wound healing by killing bacteria, promoting cell growth and providing oxygen. Notably, the cumulative release of PHMB in PBS over 48 h was 40%, while that of EGF nanoparticles was only 30%. These data indicate that individually wrapped EGF nanoparticles can avoid interaction with PHMB, but this also reduces the release rate of the EGF nanoparticles [245]. Therefore, a new therapeutic tool, platelet-rich plasma (PRP) technology, has been developed because of the presence of large amounts of GFs in platelets, which can be used for DU repair. PRP is a platelet concentrate obtained by centrifugation of a patient's fresh peripheral blood [259]. Wei et al. loaded PRP into an ODex, antimicrobial peptide-modified hyaluronic acid (HA-AMP) hydrogel and confirmed that the hydrogel can promote the proliferation and migration of L929 fibroblasts through CCK-8 assays, live/dead fluorescent staining and scratch tests. In vivo experiments showed that the hydrogel could promote wound healing in diabetic mice by regulating inflammation and accelerating angiogenesis and collagen deposition [162]. However, the high cost limits the clinical use of this technology.

### Medicines

Due to the specific pathophysiological environment of DU wounds, they are often accompanied by multiple bacterial infections, as necrotic tissues and debris can provide surfaces to which bacterial material can attach and promote the formation of bacterial biofilms, which makes the removal of microorganisms very difficult. In addition, the high levels of inflammatory factors and oxidative stress on the wound surface also hinder wound healing. In this case, the body cannot return to the normal wound healing sequence by itself, and it is necessary to assist the body by administering drugs. Yu et al. applied a skin scaffold made of PF-sodium alginate-gelatin to the wounds of diabetic rats and found that the 3% PF skin scaffold promoted wound healing by promoting collagen deposition and microvascular regeneration; in addition, this scaffold also exhibited anti-inflammatory properties [260]. Khaliq et al. prepared a cefotaxime sodium (CTX)-loaded keratin (KR)-pullulan (PL)-PVA hydrogel film dressing that was shown to accelerate wound healing in diabetic rats by reducing inflammation, recruiting fibroblasts for tissue regeneration, depositing mature collagen, and increasing angiogenesis [137]. Davani et al. developed vancomycin- and imipenem/cilastatin-loaded core shell nanofibers and proved that they can promote wound healing by significantly inhibiting the activities of *S. aureus*, methicillin-resistant *S. aureus*, *Escherichia coli*, and *P. aeruginosa* [261]. Generally, the condition of DU patients is significantly poor, and they display a decreased tolerance to drugs. Thus, how to provide an accurate dosage while avoiding the adverse reactions caused by drug accumulation is an issue worthy of exploration by researchers. Moreover, the drug resistance caused by the long-term use of antibiotics is also a problem to be solved.

### Metals

With the continuous innovations in nanotechnology, metal nanoparticles have had a substantial impact on the medical field due to their excellent chemical stability, catalytic activity, and electrical conductivity. Among them, AgNPs are widely used in the treatment of DUs due to their known antibacterial and anti-inflammatory properties, and they have been shown to act by inhibiting respiratory enzymes and disrupting microbial cell walls and DNA [262]. Tong et al. constructed a PB@PDA@AgNP composite antibacterial system that can kill bacteria by disrupting cell integrity, generating ROS, reducing ATP levels and inhibiting bacterial metabolism [263]. However, traditional industrially synthesized AgNPs are costly, complicated to generate and cytotoxic, so an increasing number of scientists are turning their attention to biosynthetic AgNPs. Ruffo et al. loaded green synthetic AgNPs into CMC hydrogels and confirmed that they possess good antioxidant, anti-inflammatory and antimicrobial activities for the treatment of DUs [244]. Krishnan et al. showed that biologically derived AgNPs reduced the mRNA and protein expression of MMP-2 and MMP-9 in wound granulation tissue and promoted early wound healing in diabetic mice [243].

In addition to AgNPs, other metal nanoparticles have also been used for the treatment of DUs. Li et al. successfully developed calcium ion crosslinked sodium alginate hydrogels containing deferoxamine and copper nanoparticles (SA-DFO/Cu), and a colony formation assay showed that these copper nanoparticles exhibited dose-dependent inhibition of E. coli and S. aureus in vitro. Moreover, DFO and copper synergistically upregulated the expression of HIF-1α and VEGF, thereby regulating angiogenesis [167]. Soledad Perez-Amodio et al. prepared poly(lactic acid) (PLA) fiber matrices loaded with calcium-releasing nanoparticles (SG5) (PLA-SG5) and experimentally demonstrated that this compound material stimulates angiogenesis, collagen synthesis, and granulation tissue formation and accelerates wound closure in the ischemic wounds of diabetic mice [264]. Of note, the metals mentioned above can avoid drug resistance and promote wound healing through different mechanisms. At present, green synthetic metals are also used in DFU treatment to improve the metal toxicity caused by traditional production methods. However, local or systemic toxicity caused by metal accumulation are still problems that we cannot avoid.

### GOx

Glucose oxidase (GOx) is an oxidoreductase with a strong deoxygenation capacity that is widely used in the food industry. As a biocatalyst, GOx can effectively catalyze the oxidation of glucose to gluconic acid and hydrogen peroxide to lower blood glucose on traumatized surfaces. Gluconic acid can lower the pH value of the trauma microenvironment and inhibit bacterial growth and hydrogen peroxide can destroy the proteins that make up the bacterial cell membrane, thus causing bacterial lysis [265–267]. Wang et al. added GOx to a polydopamine/acrylamide (PDA/AM) hydrogel to reduce trauma-mediated increase in blood glucose levels to normal. Manganese dioxide nanoparticles were also added to accelerate the decomposition of hydrogen peroxide for the production of oxygen and promotion of angiogenesis, cell crawling and cell proliferation. In addition, PDA/AM/GOx/MnO2 exhibited excellent antibacterial properties (97.87% and 99.99% against E. coli and S. aureus, respectively under 808 nm NIR radiation, and complete removal of bacterial biofilms) [247]. Shi et al. used hollow mesoporous silica nanoparticles (HMSNs) as nanocarriers to codeliver azithromycin (AZM) and GOx. The glucose consumed by GOx in the diabetic wound area effectively improved the microenvironment of chronic diabetic wounds. Moreover, the generated H2O2 effectively inhibited bacterial growth and eradicated bacterial biofilms synergistically with the effects of the antibiotic AZM [265].

All of the above applications demonstrate the great potential of GOx in the treatment of diabetic wounds, but using this enzyme also has significant drawbacks: GOx is susceptible to the high levels of proteases present in environment of DUs; large amounts of hydrogen peroxide may cause high levels of oxidative stress; and uncontrollable drug release may lead to potential long-term toxicity. To address these issues, Huang et al. encapsulated GOx in a polyacrylic acid-calcium phosphate (PAA-CaPs@Nps@GOx) hydrogel to exhibit excellent stability of enzymatic activity and controlled the degradation rate of the hydrogel by adjusting the concentration of phosphate to control the production of ROS [248].

## Other polymer dressings

**Fig. 11:**
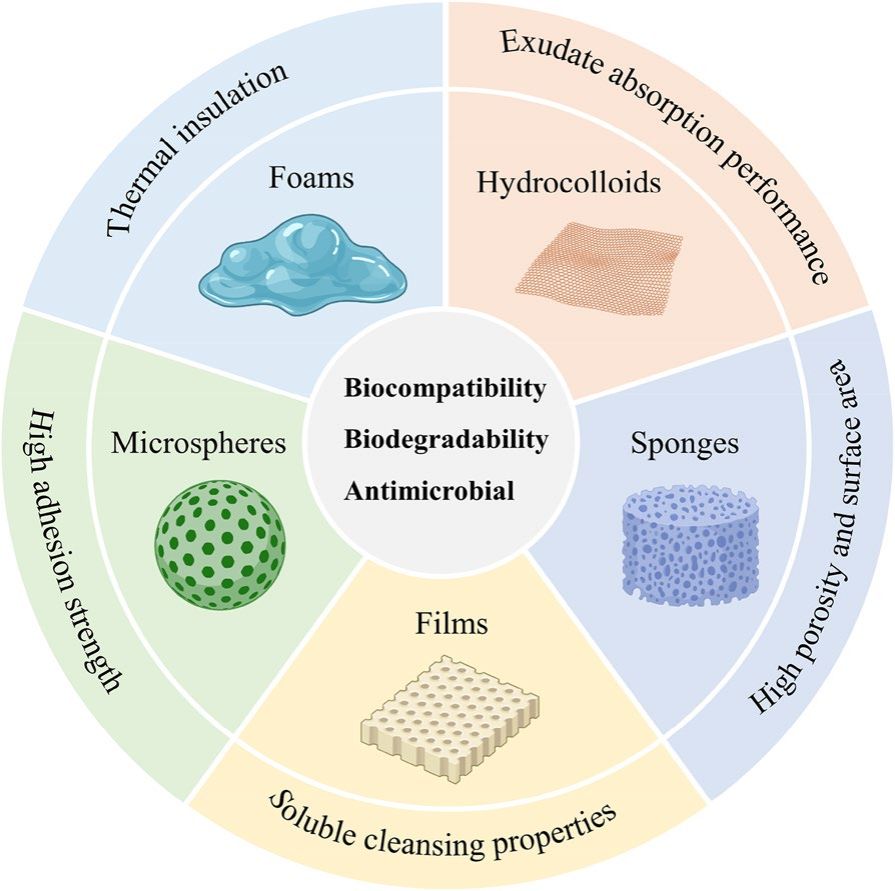
Other forms of polymer dressings and their advantages and disadvantages

To treat DUs, researchers have developed other forms of polymer dressings in addition to hydrogels, such as films, sponges, foams, hydrocolloids, and microspheres, and the observed therapeutic effects of these dressings are very encouraging (Fig. 11). In this section, we summarize the characteristics, advantages, and disadvantages of different polymeric material dressings, with the expectation of providing constructive ideas and suggestions for specifically treating DUs (Table 8).

**Table 8:** Other polymer dressings

Dressing Biomaterials	Polymer composite	Loaded substances	Animal models	Cell strains	Properties	Properties and advantages	Disadvantages	Ref.
Films	PLCL+hyaluronic	ZnO nanoparticles + oregano essential oil (OEO)	Male Sprague Dawley rats (weighing 200 ~300 g)	NIH-3T3 cells	Exchange gases; Absorbing wound exudates	Translucent; Breathable; Autolytic cleansing	Poor water absorption	[268]
	PLGA+curcumin NFMs	Heparin+ curcumin	Male fifteen-week-old SD rats (weighing 200~250 g)	Human skin fibroblast cell (HS68)	Promotes cell proliferation and migration			[269]
	Cellulose acetate+ zein	Sesamol	Male eight-week-old C57BL/6 J clean-grade mice	NA	Antioxidant; Anti-inflammatory; Release controllable			[270]
Sponges	PVA+chitosan	Pinus densiflora-iron oxide nanoparticles	NA	HEK-293 cells	Antibacterial; Hemostasis; Good permeability	High porosity and surface area; Moisture maintenance in wound area	Poor mechanical strength; Insufficient antibacterial properties	[271]
	Chitosan	Quaternary ammonium chitosan nanoparticles	Male adult Kunming mice (weighting 35 ± 3 g)	L929 cells	Bacteriostatic; Hydration; Pro-angiogenesis			[272]
	Chitosan+silk	Curcuma zedoaria +PRP-Exos	Female Sprague-Dawley (SD) rats (weighing 280 ~300 g)	NA	Easy to produce; Pro-angiogenesis; Anti-inflammatory			[273]
Hydrocolloid	Sodium Alginate	Centella asiatica (CA)	Male 6–7 weeks old Sprague-Dawley rats (weighting 250–280 g)	NA	High mechanical strength; Antibacterial	Exudate absorption; Adhesion; Autolytic cleansing	Poor adhesion; non-breathable	[274]
	Sodium Alginate-pectin	Moringa oleifera (MOL)	Male 6 to 8-week-old Sprague Dawley rats (weighing 150 ~200 g)	NA	Anti-inflammatory; Antimicrobial; Biocompatibility			[275]
	Sodium Alginate	Vicenin-2	Male 7–8 weeks old Sprague Dawley rats (weighing 150 ~250 g)	NA	Antioxidant; Anti-inflammatory;Pro-proliferation and migration of cells			[276]
Foams	Curdlan–chitosan	Ag nanoparticles	Female 5-month-old K.S.Cg Dock7 +/+Lepr /J mice ( weighting 30~40 g;)	NA	Hydration; Antimicrobial	Moisture maintenance in wound area; Shape memorability	Wound impregnation; Frequent replacement	[277]
	Polyurethanes	rhEGF	Male 6-7 weeks old SD rats	HaCaT human keratinocytes+CCD986-sk human fibroblast	Promote the proliferation of fibroblasts; Promote the formation of extracellular matrix			[278]
	HPMC/chitosan / sodium alginate +Polyurethane	Asiaticoside	Domestic farm pigs with an average (weighting 20~25 kg) and three rabbits	Human fibroblasts	Antibacterial; Hemostasis; Promoting epithelialization			[279]
Microspheres	Gelatin	rADSCs	8-weeks-old Sprague-Dawley rats (weighting 250~300 g)	HUVECs	Promote M2 macrophage polarization; angiogenesis; adhesion and proliferation	Biodegradability; Biocompatibility; Sustained-release drugs	Cumbersome preparation; Unstable embedding rate; Uncontrollable degradation rate	[280]
	PVA+chitosan grafted with phenylboric acid +celecoxib	Insulin+gelatin microspheres	Male 14-weeks old SD rats	L929 cells	Promote cell proliferation and migration			[146]
	Gelatin microspheres	Curcumin	Female BALB/c mice (weighting 18~20 g)	BJ cells and HaCat cells	Antioxidant; Anti-inflammatory; Promote cell proliferation			[281]

### Films

Films are soft elastic structures composed of transparent polymers. Films are widely used in the field of wound repair because of the following advantages: the film surface has very small pores that allow gas exchange while blocking bacterial invasion; their translucency allows changes in the wound to be observed without removing the dressing; and they are soft and fit on the skin well. Moreover, film have an autolytic debridement property, which allows them to remove crusts and necrotic tissues when treating DUs [282, 283].

Absorbency is a crucial property of dressings used for wound healing. Since most films do not have hydrophilic groups or very small pores, they display poor water absorption and are thus limited to use with mildly exuding wounds [283]. However, diabetic wounds have severe damage and large amounts of exudate. Therefore, to meet the needs of DU treatment better, researchers have prepared nanofiber films that are more easily modified with pore sizes that are easier to adjust.

Electrospinning technology is the main way by which nanofiber films are made. Streams of polymer fluids are stretched under a high voltage electrostatic field, and nanofiber films with different fiber structures, large surface areas and high mechanical strengths can be prepared by adjusting the process parameters such as voltage, flow rate, polymer concentration and solvent composition. Luo et al. fabricated nZnO/BCM films with an ideal porous structure, and their unique bead-like morphology resulted in a water vapor transmission rate of 2856.60 g/m2/day, which improved the moist environment of the damaged surface [284].

Nanofiber films are a good type of dressing to promote skin healing. It has been shown that nanofiber films, as polymers, can provide high mechanical strength and assume the role of a fibrous scaffold to provide regenerative support points for broken skin. The hematoporphyrin-doped polyketide films (Hp-PK films) made by Koo et al. provided growth attachment points for cell-dense tissues without a scaffold and transported them to the target site to promote skin regeneration. In addition, nanofiber membranes (NFMs) can improve the hydrophilicity of the film by introducing hydrophilic components, thus providing a favorable environment for skin healing [285]. Liao et al. produced PLGA/curcumin (PCH) NFMs (PCH NFMs) by grafting hydrophilic heparin molecules onto the surface of PLGA NFMs. The SEM images showed that the water contact angles of the PCH NFMs were significantly lower in all directions compared with those of the PC and PLGA films alone, demonstrating the increased hydrophilicity of the PCH NFMs. More interestingly, because of their large surface area, nanofiber films can be in full contact with the surrounding tissues and carry various components such as antimicrobial agents, GFs, and stem cells by encapsulation, adsorption, and inlaying to meet the various, specific needs of DU treatment [269]. Khan et al. prepared nanofiber films encapsulating the antimicrobial antioxidant oregano essential oil (OEO), and live-dead double-staining experiments and SEM imaging showed that in the experimental group, the E. coli colonies collapsed and 98% of the bacteria died. In vivo experiments in diabetic mice showed significant re-epithelialization of the stratum corneum, increased collagen deposition and capillary neovascularization in the treated group [268].

Recently, researchers have also linked photodynamic therapy with nanofiber films to create novel photodynamic antimicrobial dressings by carrying aggregated photosensitizers generated by highly efficient ROS with the hope of providing a new direction of thinking and exploration of killing multidrug resistant bacteria and repairing of wounds in special environments [286].

### Sponges

Sponges are substances with interconnected porous structures made by the freeze-drying method. As mentioned above, the size and number of pores are closely related to the ability to absorb exudate, and the interconnected porous structure of sponges provides the structural basis for their high water absorption. Moreover, the high porosity of sponges facilitates an adequate oxygen supply, nutrient delivery, and cell proliferation at the wound area. In addition, sponges are lightweight and feel soft on the body. Based on these advantages, sponges are widely used in the management of moderately to highly exuding wounds, such as DUs. Chitosan is the most commonly used polysaccharide in sponge manufacturing. Among the different types of polymers, the unique cationic linear structure of CS, a deacetylated form of chitin [278]. As mentioned above, the antimicrobial activity and low immunogenicity of chitosan give it the basic requirements of a dressing, while its other biological properties, such as biocompatibility, biodegradability, antioxidant activity, and in situ gelation, lay the foundation for its multifunctionality when incorporated in a dressing. However, the disadvantages of sponges, such as their poor adhesion, low mechanical strength, and insufficient antimicrobial properties, limit their practical application. Since sponges are usually nonadhesive, auxiliary dressings or tapes/bandages are required to keep them at the wound site. Adhesive aldehyde dextran sponges were prepared by Liu et al. Tissue adhesion tests showed that when the contact area between the sponge and the pig skin was approximately 18 cm^2^, the adhesion force of the sponge could reach 104 kPa, which is approximately 10 times that of fibrin glue [287].

To overcome the drawback of their low mechanical strength, researchers have enhanced the mechanical strength of sponges by crosslinking them with other polymers. Feng et al. prepared SF/KGM sponges with adjustable mechanical properties by physically crosslinking silk protein (SF) and konjac glucomannan (KGM). The stress‒strain curves showed that the addition of KGM significantly increased the tensile strength and Young's modulus of the SF/KGM sponges [288]. In addition to improving the physicochemical properties of sponges, researchers have focused on improving their biological properties in the areas of hemostasis, antibacterial activity, and healing promotion. Wei et al. prepared CS-DAC sponges from deacetylated CS and oxidized dialdehyde cellulose (DAC) via a Schiff base crosslinking reaction. The researchers found that the CS-DAC sponge efficiently induced erythrocyte and platelet aggregation and adhesion through the endogenous coagulation pathway to form a firm clot. Tail vein hemostasis experiments in mice confirmed that the CS-DAC sponge has greater hemostatic efficiency with less bleeding than the commercially available hemostatic product Celox [289]. In addition, other biomolecules (amino acids, GFs, exosomes, etc.) have been incorporated into sponges to enhance their biological properties and expand their application in the field of DU treatment [273]. In general, sponges are widely used in the treatment of DFUs due to their excellent water absorption properties and soft body feel. Although their disadvantages, such as poor adhesion, low mechanical strength and lack of antibacterial properties, limit the applications of sponges, these disadvantages can be compensated for by combining them with other materials. In the future, we expect that more substances will be used with sponge dressings to exploit their unique biological properties and provide new options for the management of DU wounds.

### Foams

Foams are wet dressings composed of polyurethane or silicone-based materials [290]. Wet dressings play a key role in healing DUs. First, wet dressings provide a moist environment. Necrotic tissue can be hydrated by the exudate and then hydrolyzed by the proteolytic enzymes secreted by the cells in the tissues for clearance. In addition, a dense and moist microenvironment creates local hypoxic tension, and this hypoxic environment promotes wound fibroblast proliferation, stimulates macrophages to release GFs, and accelerates neovascularization. Moreover, wet dressings protect the wound and isolate microorganisms in the external environment, reducing the rate of infection. Foam and sponge dressings are similar in that both have a porous structure that allows gas and water vapor penetration. They are also highly absorbent and can be used to treat high- to medium-exuding wounds [291]. However, foams have a good filling capacity and shape memory properties compared to sponges. When treating deep wounds, the foam offers protection by filling the defect and retaining its shape to keep out bacteria [292]. Available studies have shown that severe DUs can cause deep ulcers or gangrene, and properties of foam dressings would complement these applications.

Polyurethane is the most commonly used raw material for foams. Polyurethanes are synthetic polymers consisting of urethane linkages in the main chain and are formed by polymerization of isocyanates with polyols. Due to the good biocompatibility and mechanical properties of polyurethane, polyurethane-based wound dressings have become a new research hotspot [279]. The loose porous structure of polyurethane foams provides a structural basis for sustained drug release, resulting in longer lasting longer-lasting restorative effects. rhEGF-PUFS, a polyurethane foam capable of sustaining the release of recombinant human epidermal growth factor (rhEGF), was prepared by Pyun et al. and can was shown to alter the rate of wound healing by controlling the release rate of rhEGF [278]. This study lays a foundation for adaptation to meet different healing needs in the clinic. Notably, the disadvantages of foam dressings are also more obvious. If the foam dressing is too thick or too dense, it can result in reduced porosity, which in turn prevents water vapor evaporation and may lead to skin maceration around the wound [293]. Therefore, researchers need to preserve a proper porosity of the foam dressing when modifying it to allow for gas exchange. Another disadvantage of foams is that if the dressing is not changed frequently, new granulation tissue may grow into the dressing, leading to lacerations during dressing removal [294]. Unfortunately, the poor blood supply and lack of oxygen and nutrients in the DU wound result in a high risk of excessive granulation tissue proliferation. Therefore, foam dressings applied to DUs need to retain high porosity and be changed promptly.

### Hydrocolloids

Hydrocolloids are new materials made of elastic polymers, glue-forming substances such as sodium carboxymethylcellulose (NaCMC), gelatin and an adhesive that are mixed and processed, and they have shown good flexibility and ductility [295]. Hydrocolloid dressings have distinctive features. First, they are usually transparent or semitransparent, allowing for easy observation of the wound and timely dressing changes. In addition, hydrocolloids are highly adhesive and firmly adhere to the skin at the wound edge to form an airtight seal, reducing bacterial infection. Furthermore, hydrocolloids do not contain water themselves but have good water absorption properties. When in contact with wound exudate, the hydrophilic particles in the hydrocolloid form a semisolid gel that adheres to the base of the wound, providing and maintaining a moist environment conducive to healing. Moreover, because the hydrocolloid dressing contains endogenous enzymes that promote fibrinolysis and thus function to remove debris, the hydrocolloid dressing can be easily removed from the wound while avoiding secondary wound injury.

It has been reported in the literature that the keys to DU management are debridement, frequent dressing changes and a moist healing environment. Based on the advantages mentioned above, hydrocolloids are widely used in the treatment of DUs. Tan et al. prepared a hydrocolloid film by combining the anti-inflammatory antioxidant Vicenin-2 (VCN-2) with sodium alginate (SA). Studies based on the STZ-induced diabetic rat model have shown that VCN-2-containing hydrocolloid membranes can increase the expression of VEGF and TGF-β in a hyperglycemic environment, thereby promoting cell proliferation, migration, and wound healing. Interestingly, VCN-2-containing hydrocolloid films applied topically to diabetic wounds also increased host insulin levels, although the exact mechanism needs to be further investigated [276]. To further expand the applications of hydrocolloids, researchers have added natural or synthetic polymers to improve their swelling, adhesion and mechanical strength. Jin et al. developed a novel *Centella asiatica* (CA)-loaded hydrocolloid dressing (HCD). The results of the mechanical property experiments showed that crosslinked sodium CMC significantly improved the swelling rate of CA-loaded HCDs, while the addition of petroleum resin hydrocarbon (PHR) increased their mechanical strength and flexibility [274].

Due to the limited water absorption properties of natural materials alone, researchers have prepared hydrocolloids by combining various absorbent substances for use in hyperosmolar wounds such as DUs. Wojcik et al. made a highly absorbent hydrocolloid based on curdlan and a highly absorbent Kydex material in combination with agarose and chitosan. The absorption tests showed that this dressing had an excellent ability to absorb exudate, with 1 g of dressing absorbing approximately 15 ml of exudate. Another experiment demonstrated that the maximum absorption capacity (Cmax) of this dressing to serum and plasma reached 1563±57% and 1202±140%, respectively [296]. Of note, the main disadvantage of hydrocolloids is that when the hydrophilic fillers dissolve, swell and exude, the surface energy of the hydrocolloid dressing increases dramatically. When the surface energy of the dressing is much higher than that of human skin, its adhesion decreases and is eventually lost, leading to dressing dislodgement [295]. This will remind future researchers to pay attention to the exudation of the hydrophilic fillers in hydrocolloids, as well as the water contact angle and surface energy after a sufficient amount of water is absorbed, to develop new hydrocolloid dressings with controlled exudation and continuous self-adhesion.

### Microspheres

Microspheres are very small spheres or sphere-like bodies with a particle size generally in the range of 1 to 250 μm that are formed by dissolving or uniformly dispersing a drug in a polymeric material. Many techniques are used to prepare microspheres, and the appropriate method needs to be chosen according to the physicochemical properties of the drug and the desired application of the microspheres [297]. The commonly used methods are emulsification and volatilization, crosslinking, phase separation, spray drying, and hot-melt extrusion. Biopolymer microspheres are common organic polymer microspheres that are mainly prepared from proteins (gelatin, albumin, silk protein, etc.) and polysaccharides (gum arabic, alginate, chitosan, etc.) and have the advantages of stability, nontoxicity, good sphericity, biocompatibility, degradability, and no toxic side effects from the degradation products. Microspheres have been widely used in the field of DUs. Liu et al. prepared neurotensin (NT)-loaded gelatin microsphere dressings (NT/GMs/SF dressings) with silk protein (SF) as a scaffold. Wound experiments in diabetic rats showed that wound repair with the NT/GMs/SF dressings was significantly faster than that observed in the control group [298]. Another research team, Liu et al. prepared gelatin microspheres (CNPs@GMs) encapsulated by curcumin (Cur) nanoparticles. In vivo experiments in diabetic mice showed that CNPs@GMs promoted cell migration to the trauma surface and neovascularization [281].

In diabetic wounds, hypoxia is a major factor affecting cell survival, migration and vascularization. In a recent study, Guan et al. developed a novel dressing (Gel/ORM) consisting of a combination of oxygen-releasing microspheres (ORMs) that continuously produce oxygen and a ROS-scavenging hydrogel. These microspheres can continuously convert H_2_O_2_ into oxygen by binding to catalase. In vivo experiments in diabetic mice demonstrated that the oxygenation and ROS scavenging observed in the Gel/ORM group increased cell survival, accelerated cell migration, stimulated angiogenesis, and reduced oxidative stress and inflammation compared to the nontreated and gel groups. Furthermore, Gel/ORM provides a novel therapeutic solution in the exploration of accelerating chronic diabetic wound healing without the use of drugs [299]. However, the disadvantages of microsphere dressings are their cumbersome preparation process, high production costs, great cytotoxicity, unpredictable encapsulation rate and uncontrollable degradation rate. At present, it seems that the application of microsphere dressings for the treatment of DUs still has many problems. However, researchers are currently overcoming these drawbacks by simplifying the process, chemically modifying the microspheres, and combining the microspheres with other polymeric materials. It is believed that with continuous research on microsphere materials, in the future, microspheres will play an indispensable role in the field of DUs.

## Conclusion and prospects

Healing diabetic wounds is challenging due to their complex pathophysiological mechanisms. This paper reviews various additives (stem cells, exosomes, drugs, metal, GOx) and summarizes the recent advances in various hydrophilic polymeric hydrogels (chitosan, alginate, HA, cellulose, gelatin) that are currently used for DU treatment.

From the literature discussed in this paper, almost all animal experiments have remained at the level of rodents. Although our current research data are not sufficient to support experiments in humans, we could try to carry out experiments on animals (such as pigs) that have skin that is more similar to human skin to lay a foundation for subsequent clinical experiments. Second, most of the studies have focused on only phenotype (such as anti-inflammatory, angiogenic, antioxidant, etc.), but not on specific targets, molecular mechanisms of action, or kinetic reactions. Third, many researchers are currently often adding drugs to hydrogels to obtain better therapeutic effects but do not pay much attention to their pharmacokinetic or pharmacokinetic parameters, which results in sudden release effects and high local drug concentrations, thus affecting experimental safety. Fourth, the microenvironment of DUs is complex and diverse, and even the same patient may display great differences in disease progression processes. Therefore, we should implement individualized strategies and process strategies and adjust our treatment plans in a timely manner. Fifth, intelligent and multifunctional hydrogels could also be studied. Sixth, although most of the hydrogels that have been recently studied in the laboratory claim to have multiple functional properties, in practice, it has been found that it is often difficult to obtain good overall wound closure compared to the normal wound healing process, mainly due to the interactions between multiple associated factors in difficult-to-heal wounds. How to solve this problem is also one of the future research directions for scientists.

In summary, research on hydrogels used to treat DUs is developing rapidly. Although there their application is still a certain distance from clinical practice, it is believed that with the concerted efforts of experts, hydrogels will be applied clinically in the near future.

## Data Availability

Not applicable.

## Abbreviations

NA: Not applicable or not available
EGF: Epidermal growth factor
BMMNCs: Bone marrow mononuclear cells
HUVECs: Human umbilical vein endothelial cells
HUMSCs: Human umbilical cord mesenchymal stem cell
PRP: Platelet-rich plasma
bFGF: Basic fibroblast growth factor
HDFs: Human dermal fibroblasts
CS-DA-LAG: Chistoan- dihydrocaffeic acid- L-arginine
PEG-PBA-BA: Polyethylene glycol-phenylboronic acid- formylbenzoic acid
PBA: Phenylboronic acid
PVA: Polyvinyl alcohol
HAMA: Hyaluronic acid methacrylate
VEGF: Vascular endothelial growth factor
GelMA: Gelatin methacryloyl
CONPs: Cerium oxide nanoparticles
SNAP: S-Nitroso-N-acetylpenicillamine
VAN-AgNCs: Vancomycin-conjugated silver nanoclusters
SCS: Succinyl chitosan
HA-ALD: Hyaluronic acid-aldehyde
CMCS: Carboxymethyl chitosan
OHA: Oxidized hyaluronic acid
OHMPC: Oxidized hydroxymethyl propyl cellulose
HA-ADH: Adipic dihydrazide-modified hyaluronic acid
SA: Sodium alginate
CMCS-Hep: N, O-carboxymethyl chitosan-heparin
CMC-A: Carboxymethyl cellulose-aldehyde
BMMSC-Exo: Bone marrow mesenchymal stem cell-derived exosome
Alg@ori: Oridonin loaded alginate microspheres
HA-PEI@siRNA-29a: siRNA-29a gene-loading hyaluronic acid-polyethyleneimine complex
AD-MSCs: Adipose-derived mesenchymal stem cells
ADSCs: Adipose-derived stem cells
HF-MSCs: Hair follicle‑derived Mesenchymal stromal cells
HDF: Human dermal fibroblasts
ESCs-Exo: Epidermal stem cells derived exosomes
EPC: Endothelial progenitor cells
aFGF: Acidic fibroblast growth factor
SCF: Stem cell factor
PDGF-BB: Platelet derived growth factor-BB
DCH: Doxycycline hyclate
CEX: Cephalexin
GOx: Glucose oxidase
DFO: Deferoxamine mesylate
rhEGF: Recombinant human epidermal growth factor
PLGA: Poly (lactic-co-glycolic acid)
HPMC: Hydroxypropyl methylcellulo
